# Transcription Factor E2F4 Promote Proliferation, Migration, and Invasion of Gastric Cancer Cells by transcriptionally activating DSCC1

**DOI:** 10.7150/ijbs.99590

**Published:** 2024-09-16

**Authors:** Shantanu Baral, Yantao Yu, Qiannan Sun, Mingrui Jiang, Ruiqi Li, Yifan Cheng, Arawker Mubeen Hussein, Youquan Shi, Yongjun Jiang, Dong Tang, Sen Wang, Daorong Wang

**Affiliations:** 1 Northern Jiangsu People's Hospital Affiliated to Yangzhou University, Yangzhou, Jiangsu, 225001, P. R. China.; 2 Northern Jiangsu People's Hospital, Yangzhou, Jiangsu, 225001, P. R. China.; 3 General Surgery Institute of Yangzhou, Yangzhou University, Yangzhou, Jiangsu, 225001, P. R. China.; 4 Yangzhou Key Laboratory of Basic and Clinical Transformation of Digestive and Metabolic Diseases, Yangzhou, Jiangsu, 225001, P. R. China.; 5 Northern Jiangsu People's Hospital, Clinical Teaching Hospital of Medical School, Nanjing University, Yangzhou, Jiangsu, 225001, P. R. China.; 6 The Yangzhou School of Clinical Medicine of Dalian Medical University. Yangzhou, Jiangsu, 225001, P. R. China.; 7 Department of General Surgery, The First Affiliated Hospital of Nanjing Medical University, Nanjing, Jiangsu, 210029, P. R. China.

**Keywords:** Gastric Cancer, DNA Replication and Sister Chromatid Cohesion 1, Early 2 Factor 4, transcription, Proliferation

## Abstract

Gastric cancer (GC) ranks as the fifth most common cancer and the fourth leading cause of cancer-related deaths globally. Despite advancements in molecular profiling, the mechanisms driving GC proliferation and metastasis remain unclear. This study identifies Early 2 Factor 4 (E2F4) as a key transcription factor that promotes GC cell proliferation, migration, and invasion by upregulating DNA Replication and Sister Chromatid Cohesion 1 (DSCC1) expression. Bioinformatics and transcription factor analyses revealed E2F4 as a significant regulator of DSCC1. Functional assays confirmed E2F4's role in enhancing GC cell malignancy *in vitro* and *in vivo*. Knockdown and overexpression experiments demonstrated that E2F4 positively regulates DSCC1 at the transcriptional level, with ChIP-qPCR and dual luciferase reporter assays validating the binding sites on the DSCC1 promoter. These findings highlight the E2F4-DSCC1 axis as a potential therapeutic target to mitigate GC progression.

## Introduction

Gastric cancer (GC) is a profoundly deadly disease on a global scale, standing as the fifth most commonly detected cancer and the fourth leading contributor to cancer-related mortality internationally^1^. In recent times, advancements in high-throughput and genomic technologies have enabled the study of gastric cancers at the molecular level. The availability of molecular profiling data has significantly aided in pinpointing potential gene driver alterations in GC, including gene mutations, chromosomal changes, alterations in transcription patterns, and irregularities in epigenetic modifications^2^-^4^. Therefore, comprehending the process of GC metastasis and pinpointing molecular markers and therapeutic targets associated with metastasis becomes imperative.

Cell proliferation, migration, and invasion are essential biological processes vital for both normal physiological functions and disease states such as cancer. These dynamic cellular activities are integral to tissue development, wound healing, immune responses, and the spread of cancerous cells. When these processes become dysregulated, they fuel tumor progression and metastasis across various cancer types, rendering them attractive targets for therapeutic intervention^5^-^7^. A comprehensive understanding of the molecular mechanisms governing these cellular behaviors is crucial for the development of effective cancer treatments. Multiple signaling pathways and molecular components participate in orchestrating these processes, including those governing cell cycle regulation, epithelial-mesenchymal transition (EMT), and remodeling of the extracellular matrix (ECM)^8^-^10^. Investigating the intricate interplay between these pathways and their perturbations in cancer cells provides valuable insights into cancer biology and unveils potential therapeutic avenues.

DNA Replication and Sister Chromatid Cohesion 1 (DSCC1, also referred to as DCC1) is a key molecular component in the formation of the Chromosome Transmission-Fidelity Protein 18 (CTF18)-DSCC1-CTF8 (CTF18-1-8) module. DSCC1 is primarily localized within the nucleus of cells^11^. It exhibits a strong correlation with the proliferation and metastasis of colon or prostate cancer cells^12^-^14^. This protein complex is instrumental in various cellular processes including sister chromatid cohesion, DNA replication, spindle checkpoints, DNA repair, and maintenance of genome stability during the S phase of the cell cycle. Numerous studies have highlighted its role in promoting cell proliferation and its association with unfavourable prognosis in hepatocellular carcinoma^15^-^17^. However, no research has yet explored the involvement of DSCC1 in gastric cancer or the significance of molecular systems in this context.

Early 2 Factor 4 (E2F4) gene encodes a transcription factor crucial for regulating cell cycle advancement and proliferation. As a member of the E2F family, E2F4 intricately manages genes vital for DNA synthesis and cell division^18^. Its aberrant activity has been linked to diverse human cancers like breast, lung, and bladder cancers^19^-^21^. Understanding E2F4's function and regulation is pivotal for deciphering the molecular intricacies of tumorigenesis and devising targeted cancer treatments. Nevertheless, the association of E2F4 with GC and its impact on GC cell proliferation have seldom been explored in research.

In this research, the relationship between DSCC1 and the proliferation of GC cells was investigated. E2F4 was identified as a transcription factor regulating DSCC1 expression. It was validated that E2F4 activated the cell cycle pathway in a DSCC1-dependent manner, consequently amplifying the proliferation, migration, and invasion capacities of GC cells.

## Materials and Methods

### Bioinformatic analysis

To identify genes associated with GC in humans, we searched the GEO database (www.ncbi.nlm.nih.gov/geo/) for expression profiling by array datasets. We retrieved five microarray datasets (GSE103236, GSE118916, GSE79973, GSE112369, and GSE26899) for analysis. The affy package in R was utilized for microarray dataset normalization and background noise removal. Subsequently, the limma package was employed to identify differentially expressed mRNAs (DEGs) in GC using a t-test with a significance threshold of P-value < 0.05 and logFC^22^, ^23^. The MCODE plugin was used to identify hub genes from the clusters of DEGs^24^. Transcription factors (TFs) were predicted from the protein-protein interaction (PPI) network using the iRegulon plugin in Cytoscape^25^. The regulatory network was constructed and visualized using Cytoscape. We conducted functional enrichment analyses of hub genes using Enrichr for gene ontology (GO) and Kyoto Encyclopedia of Genes and Genomes (KEGG) pathways^26^. Additionally, we obtained Hallmark gene sets for gene set enrichment analysis (GSEA) and classified samples into high and low groups based on the median expression levels of DSCC1 and E2F4 genes. Upregulated and downregulated genes were determined by comparing differences in gene expression levels between the two groups. Gene sets with | NES |> 1 and FDR < 0.05 were regarded as significant. Additionally, three machine learning algorithms (SVM-REF, randomForest, and LASSO) were used to analyze hub genes, and common genes identified by all three algorithms were considered significant for GC. Expression profiles and protein levels of DSCC1 and E2F4 in stomach adenocarcinoma (STAD) were obtained from the TCGA database, and patient survival analysis was conducted using the KaplanMeier plotter^27^. STAD gene mutation data and RNA-seq data were also acquired from the TCGA database. GenVisR data package was utilized to construct and visualize the "waterfall map" of STAD gene mutations^28^. The UCSC Genome Browser was employed to search for DSCC1 promoter sequences and analyze transcription factors regulating DSCC1^29^. Pearson correlation analysis between DSCC1 expression and its transcription factor E2F4 was performed using the "ggstatsplot" and "ggplot2" packages. Finally, the JASPAR database was consulted to identify the binding site of transcription factor E2F4 to the DSCC1 promoter^30^.

### Clinical samples

Human surgical specimens, including formalin-fixed paraffin-embedded specimens of GC (n=80, aged 43 to 77 years) and freshly excised GC tissues (n=16; comprising 8 males and 8 females, aged 46 to 69 years), were obtained from Northern Jiangsu People's Hospital Affiliated to Yangzhou University (Yangzhou, China) between 2021 and 2023. None of the patients received preoperative chemotherapy. Two qualified pathologists confirmed the GC diagnosis. Disease staging was determined according to the American Joint Committee on Cancer TNM staging system (AJCC-8 TNM). Clinicopathological data including age, sex, tumor size, TNM stage, degree of differentiation, histological grade, venous and nerve invasion were collected from hospital records. The study was approved by The Northern Jiangsu People's Hospital Affiliated to Yangzhou University, and the Ethics Committee of Yangzhou University. All procedures were conducted in accordance with the ethical standards of the institutional research committee and the Declaration of Helsinki. Written informed consent was obtained from each patient prior to sample collection.

### Real-time quantitative PCR (RT-qPCR)

Total RNA extraction from GC cells and tissue samples was carried out using Trizol reagent (Vazyme, Nanjing, China) following the manufacturer's instructions. Subsequently, reverse transcription of the total RNA was conducted using a reverse transcription kit (Vazyme, Nanjing, China). Quantitative analysis was performed utilizing SYBR Green PCR Master Mix (Yeasen, Shanghai, China) on a Applied Biosystems StepOnePlus No.4376600 (Applied Biosystems, Waltham, MA, USA). GAPDH served as an internal reference gene for normalization. Fold changes were determined using the 2-ΔΔCt method. All experiments were replicated in triplicate. Primer sequences are detailed in **Supplementary Table 1**.

### Immunohistochemistry (IHC)

The process involved in handling GC tissue samples included fixation, embedding, slicing, dewaxing, antigen retrieval, primary antibody incubation, secondary antibody incubation, DAB staining, hematoxylin staining, and alcohol denaturation. Primary antibodies used were anti-DSCC1 (1:50, Abmart, PHU7133) and anti-E2F4 (1:100, Bioss, bs-1399R). Slides were examined and imaged with an Olympus BX53 fluorescent microscope (Olympus Corporation), and staining for DSCC1 and E2F4 was assessed by cell positivity and intensity. Staining intensity was graded from 0 to 3 (colorless to dark brown), while the percentage of positive cells was scored from 0 to 4 (0-5% to >75%). The final score was derived from a combination of staining intensity and the percentage of positive cells. Cases with a score of ≥5 were categorized as having "high expression," while those with a score of ≤4 were considered to have "low expression".

### Western blot

Total protein from GC cells and tissue samples was extracted using radio immunoprecipitation assay (RIPA, Solarbio, Beijing, China). Following extraction, total protein was separated on 10-12% SDS/PAGE gels and transferred onto polyvinylidene difluoride (PVDF) membranes (Millipore, Boston, MA, USA). Subsequently, membranes were blocked using 5% skim milk and then incubated overnight at 4 °C with primary antibodies. The following day, PVDF membranes were incubated for 1.5 hours at room temperature with suitable secondary antibodies, and ECL substrate was applied. Images were captured using a Tianneng automatic chemiluminescence image processing system (TANON, Shanghai, China). Primary antibodies are listed in **Supplementary Table 2**. The Western blot bands' intensity was measured using ImageJ software (version 1.51; National Institutes of Health).

### Cell culture and drug treatment

GC cell lines AGS, NCI-N87, HGC-27, and BGC-823, along with the gastric mucosal normal epithelial cell line GES-1, were procured from the Cell Bank of Type Culture Collection of Chinese Academy of Sciences, Shanghai Institute of Cell Biology, Chinese Academy of Sciences. These cell lines were cultured in Roswell Park Memorial Institute (RPMI) 1640 medium (Gibco, Waltham, MA, USA) supplemented with 10% fetal bovine serum (FBS; Gibco) and 1% penicillin-streptomycin (Solarbio, Beijing, China) under standard conditions of 37 °C temperature, 5% CO2, and humidified atmosphere. Palbociclib (MCE, HY-50767; Monmouth Junction, USA) and HLM006474 (MCE, HY-16667; Monmouth Junction, USA) were dissolved in DMSO and added to the culture medium at the designated concentration. The cells were subsequently exposed to the drugs for 2 days at 37°C, unless otherwise indicated.

### Cell transfection

Small hairpin RNA (shRNA) directed against DSCC1 and E2F4, along with a negative control shRNA (sh-Ctrl), were synthesized by General Boil (Shanghai, China). The coding sequence of DSCC1 or E2F4 was amplified and inserted into the pCDH-CMV-MCS-EF1-copGFP plasmid (General Boil) and then packaged into lentivirus. The lentivirus was transfected into selected GC cell lines. Positive cells were selected using puromycin (2 μg/mL) for two weeks, and the efficacy of knockdown and overexpression was assessed via RT-qPCR and western blotting.

### Cell proliferation assay

The cell counting kit 8 (CCK-8, Yeasen, Shanghai, China) was employed to assess the proliferation capacity of GC cells following the manufacturer's protocol. Cells from both the sh-Ctrl and shRNA groups were seeded into 96-well plates (Corning, USA) at a density of 1×10^3^ cells per well. At 0h, 24h, 48h, 72h, and 96h time points, 10 μL of CCK8 solution was added to each well and incubated for 2 hours at 37 °C to evaluate proliferation. The absorbance was then measured at 450 nm using a BioTek microplate reader (BioTek, Winooski, VT). The Colony formation assay was conducted in 6-well plates (Corning). 5x10^2^ cells from various experimental groups were seeded into each well and incubated at 37°C for 14 days. After incubation, colonies were fixed with 4% paraformaldehyde for 10 minutes and then stained with 5% crystal violet solution (Solarbio, Beijing, China) for 5 minutes. The number of visible colonies (comprising >50 cells) was manually counted from three independent replicates after the plates had dried.

### EdU assay

An EdU assay was conducted utilizing an EdU assay kit (Beyotime, Shanghai, China). Cells were exposed to a 10 μM EdU solution at room temperature for 2 hours. Following this, the cells were fixed with 4% neutral paraformaldehyde at room temperature for 30 minutes. Subsequently, cell permeabilization was achieved using PBS containing 0.5% Triton X-100 for 20 minutes, followed by three washes with PBS. The cells were then stained using the BeyoClick EdU Assay kit (Beyotime, Shanghai, China) for 30 minutes and washed three times with washing solution. Finally, cell nuclei were counterstained with Hoechst 33342 (Beyotime, Shanghai, China) and observed under a microscope***.***

### Transwell assay

Transwell chambers (Corning, USA) were utilized to evaluate cell migratory capacity. Cells were suspended in RPMI 1640 medium devoid of FBS, and 1×10^4^ cells were seeded into the upper chamber of the Transwell insert. Subsequently, 500 μL of RPMI 1640 medium supplemented with 10% FBS was added to the lower chamber. After 24 hours, cells that had migrated to the lower surface of the membranes were fixed with 4% paraformaldehyde for 10 minutes and stained with 5% crystalline violet solution (Solarbio, Beijing, China) for 5 minutes. The average number of migrated cells was determined by examining six randomly chosen fields of view under the microscope.

### Wound healing assay

The wound healing assay was conducted using 6-well plates (Corning, USA). Cells from different experimental groups were seeded into the wells and allowed to grow until reaching 90% confluence. A scratch was created using a pipette tip, followed by washing away the detached cells with PBS. Subsequently, cells were cultured in RPMI 1640 medium without FBS for 24 hours. The width of the wound was measured at 0 hours, 24 hours, and 48 hours using a light microscope, and ImageJ software (version 1.51; National Institutes of Health) was utilized to quantify the relative area of wound closure.

### Cell cycle assay

Cell cycle analysis was conducted using flow cytometry. Cells were initially washed twice with PBS and then treated with trypsin, followed by a 4-hour exposure to 70% ethanol at 24°C. Afterward, the cells were incubated in 500 μL of a prepared propidium iodide (PI) staining solution (Yeasen, Shanghai, China) at 37°C for 30 minutes, and then analysed using a flow cytometer (BD LSRFortessa; BD Biosciences, Franklin Lakes, USA). The resulting data were analyzed using FlowJo software (v10.8.1).

### Immunofluorescence (IF)

Cells cultured on confocal culture dishes (NEST, Wuxi, China) were fixed using 4% paraformaldehyde and permeabilized with 0.01% Triton X-100 for 15 minutes. Subsequently, the dishes were treated with primary antibodies, anti-DSCC1 (1:100, Bioss, bs-7720R) or anti-E2F4 (1:100, Bioss, bs-1399R), overnight in a humidified chamber at 37 °C in the dark. Following this, cell nuclei were stained with DAPI. The fluorescence distribution and intensity of the cells were then assessed using a Zeiss LSM 880 laser microscope (Carl Zeiss AG, Oberkochen, Germany).

### Chromatin-immunoprecipitation (ChIP)

ChIP assays were performed using the SimpleChIP Enzymatic Chromatin IP Kit (CST, Massachusetts, MA, USA). Cells were fixed with 1% paraformaldehyde for 10 minutes, followed by addition of 0.125 M glycine to stop DNA-protein crosslinking at room temperature for 5 minutes. SDS lysis buffer containing protease inhibitors was used to lyse the cells, and chromatin fragments were generated using an ultrasonic fragmentation device. A portion of the lysates was designated as “Input.” The remaining lysates were incubated with E2F4 antibody (CST, #40291) and Protein G magnetic beads to form a DNA-antibody-magnetic bead complex. After elution and purification, the DNA was labelled as “Target.” Rabbit IgG (CST, Massachusetts, MA, USA) was used as a negative control. The final purified DNA fragments were analysed using RT-qPCR. Primer sequences for qPCR targeting the DSCC1 promoter binding site are provided in **Supplementary Table 3**.

### Luciferase activity assay

The luciferase activity assay was conducted following the protocol outlined in the Dual Luciferase Reporter Gene Assay Kit (Yeasen, Shanghai, China). Initially, the wild-type (WT) and mutated DSCC1 UTR were inserted into the PGL3-Basic Vector to generate vecE2F4+DSCC1Wt and vecE2F4+DSCC1Mut constructs. Subsequently, 293T cells were co-transfected with various plasmids for 48 hours. Finally, luciferase activity was measured using the Synergy LX (BioTek) instrument.

### Co-immunoprecipitation (co-IP) assay

The Co-IP assay was conducted following previously established protocols^31^. Lysates were mixed with Flag affinity beads (Sigma-Aldrich, MA, USA), and the interacting proteins were identified using western blot analysis.

### Xenograft assay

NCI-N87 cells were transfected with shRNA targeting DSCC1, E2F4, and corresponding scramble controls via lentiviral infection, with each plasmid tagged with luciferase. The cells, suspended in 100 µl PBS, were then subcutaneously injected into the left armpit of female BALB/c nude mice aged 4-5 weeks (Ethics Committee for Animal Experiments of Yangzhou University). To evaluate the combined effects of E2F4 inhibitor HLM006474 (20 mg/kg) and cell cycle pathway inhibitor Palbociclib (2 mg/kg) on NCI-N87-induced tumors *in vivo*, 1×106 cells were injected subcutaneously into the mice. Tumor volumes were measured every 7 days using the formula (length × width^2)/2. After 28 days, the mice were euthanized by cervical dislocation under anesthesia, following Laboratory Animal Guidelines for Ethical Review of Animal Welfare^32^. Anesthesia was induced by CO2 asphyxiation, and death was confirmed by dilated pupils. Tumors were removed and weighed at the end of the experiment.

### Statistical analysis

Functional analysis was conducted using the SPSS software package (version 24.0, IBM SPSS, IL, USA) and GraphPad Prism (version 8.4.3, GraphPad Software, CA, USA). Student's t-test was employed to compare means between two groups, while two-tailed ANOVA was utilized for comparisons across multiple groups. The Chi-square test was applied for correlation analysis between gene expression and clinicopathological features. Additionally, the Kaplan-Meier method was utilized to assess overall survival differences among various groups. Cox regression analysis was employed to identify factors influencing patient prognosis. Statistical significance was set at a p-value < 0.05.

## Results

### Comprehensive analysis reveals key hub genes in GC

**Figure 1:**
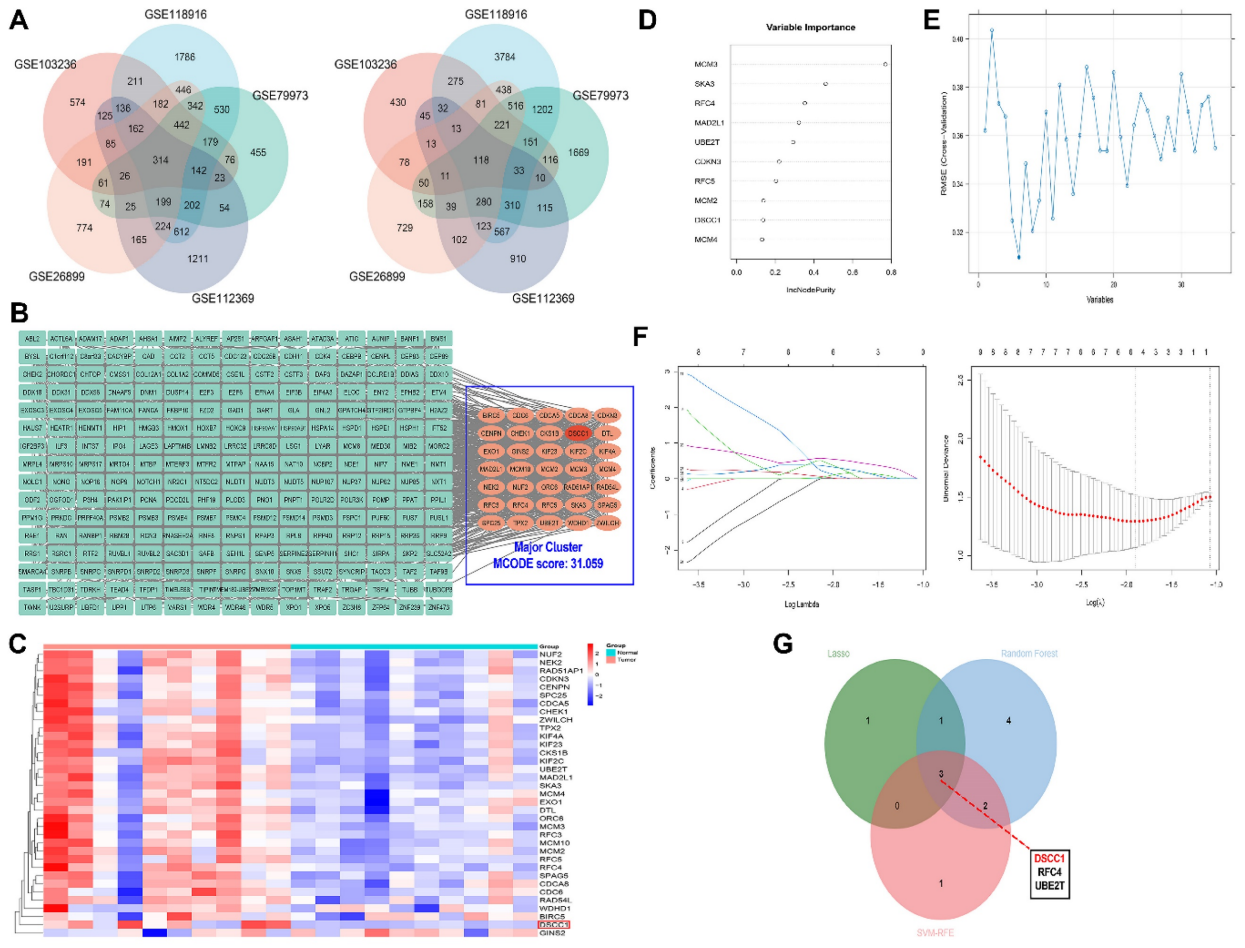
** Identification and Prognostic Analysis of Hub Genes in Gastric Cancer. (A)** Identification of common DEGs using GEO datasets.** (B)** Interaction network of differentially expressed upregulated mRNAs. **(C)** Heatmap showing the expression of 35 hub genes in GC and normal samples. **(D)** Machine learning and hub-gene screening through SVM-REF analysis.** (E)** randomForest analysis. **(F)** LASSO analysis. **(G)** Identified hub genes.

Five GEO datasets (GSE1032326, GSE118916, GSE79973, GSE112369, and GSE26899) were independently analyzed to identify DEGs in GC. All identified DEGs were statistically significant based on P-values. To mitigate sample bias and exclude the effects of genetic conditions other than GC, we focused on the common DEGs across all datasets. A total of 432 common DEGs were identified, including 314 upregulated and 118 downregulated genes **(Figure 1A)**. The PPI network of the 314 upregulated genes consisted of 275 nodes and 2654 edges. Cluster analysis using MCODE identified 12 clusters, with the major cluster **(Figure 1B)** having the highest MCODE score of 31.059. This primary cluster comprised 35 nodes and 528 edges. We generated a heatmap of these 35 hub genes to compare their expression between gastric tumor samples and normal tissues **(Figure 1C)**. Using Enrichr, we conducted GO and pathway enrichment analysis of the hub genes. We identified 374 enriched GO terms under Biological Process, 75 under Molecular Function, and 33 under Cellular Component. The top 10 most significant GO enriched terms are presented in **(Supplementary Figures 1)**. Our findings indicate that the upregulated hub genes are primarily involved in biological processes such as ribosome biogenesis, DNA metabolic processes, and rRNA processing. In the Molecular Function category, RNA binding, DNA replication origin binding, and snoRNA binding were the most significantly enriched terms. Within the Cellular Component category, nuclear lumen, intracellular non-membrane-bound organelles, and nucleolus were the key enriched terms. Additionally, Enrichr identified 24 enriched KEGG pathways, with 5 pathways showing P<0.05, as detailed in **(Supplementary Table 4)**. To further identify the key hub genes in GC, we applied three machine learning algorithms: SVM-REF, random forest, and LASSO. We selected the common genes based on the top 6 genes from SVM-REF, the top 10 genes from random forest, and the results from LASSO **(Figure 1D-G)**.

### Elevated DSCC1 expression correlates with poor prognosis in GC

**Figure 2:**
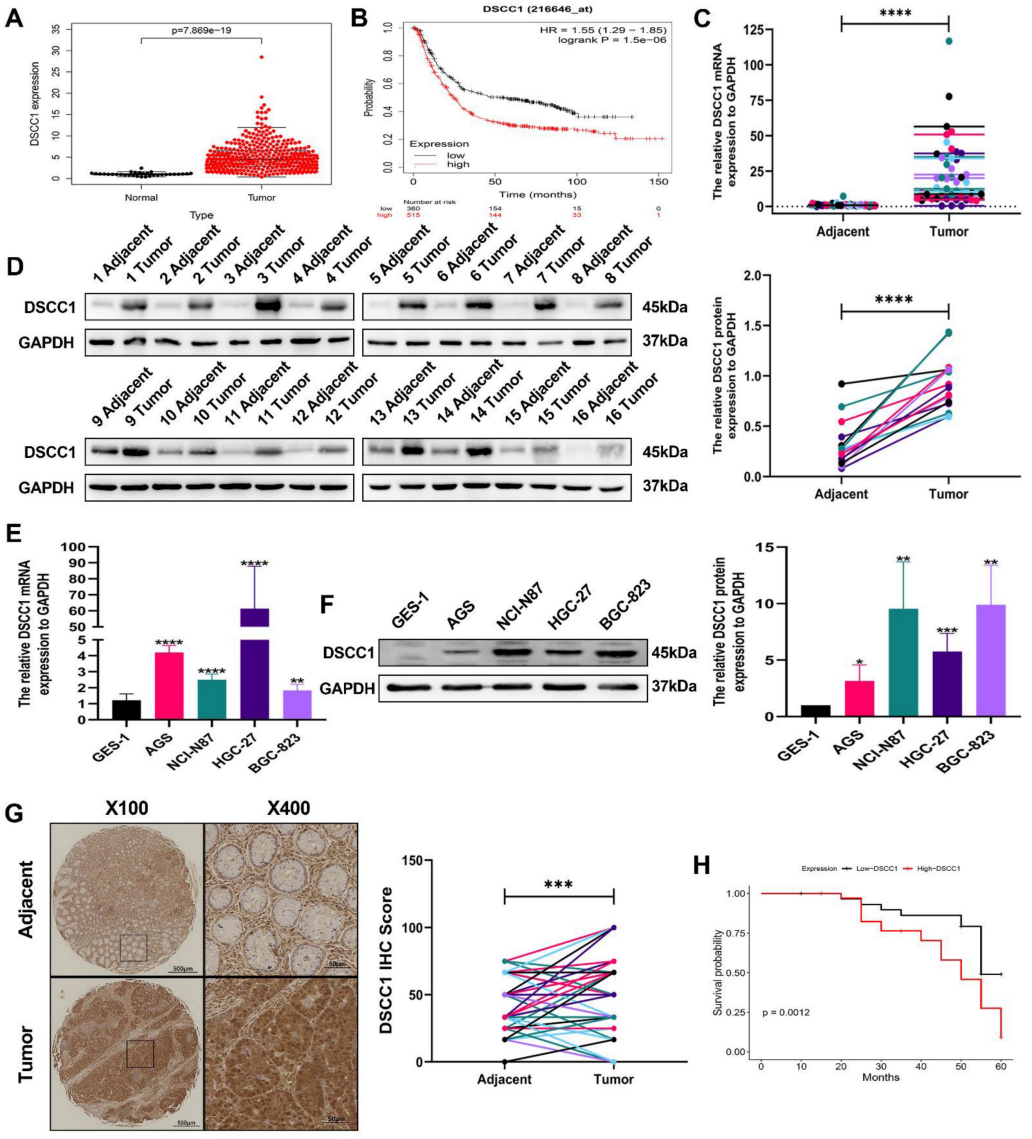
** Up-regulation of DSCC1 in GC tissues and cells. (A)** Comparison of DSCC1 expression between tumor tissues and adjacent tissues from the TCGA database. **(B)** Comparison of DSCC1 expression levels between high and low expression groups from the Kaplan-Meier Plotter. **(C)** Detection of DSCC1 mRNA expression in 16 pairs of GC and corresponding adjacent tissues using RT-qPCR. **(D)** Detection of DSCC1 protein expression in 16 pairs of GC and corresponding adjacent tissues using Western blot. **(E)** Analysis of DSCC1 mRNA and protein expression in GES-1 and GC cell lines by RT-qPCR. **(F)** Analysis of DSCC1 mRNA and protein expression in GES-1 and GC cell lines by Western blot.** (G)** Detection of DSCC1 expression in 80 paired GC and adjacent non-tumor tissues by IHC. **(H)** Survival analysis of GC patients with varying levels of DSCC1 expression. (*P<0.05, **P<0.01, ***P<0.001, ****P<0.0001) - Significance levels denoted by asterisks.

To validate the expression of the DSCC1 gene and its relationship with overall survival in STAD patients, we used data from the TCGA database and the Kaplan-Meier plotter for analysis. The correlation between DSCC1 expression levels and overall survival in STAD patients is presented in **(Figure 2A-B)**. The analysis revealed a significant difference in DSCC1 expression levels between STAD samples and normal tissues. Additionally, RT-qPCR and western blot experiments confirmed increased DSCC1 RNA and protein levels in 16 pairs of fresh specimens from GC patients, showing significantly higher DSCC1 expression in GC tissues **(Figure 2C-D)**. Western blot analysis and qRT-PCR of various cell lines further validated the increased DSCC1 expression in four GC cell lines (AGS, NCI-N87, HGC-27, and BGC-823) compared to the normal cell line GES-1 **(Figure 2E-F)**. IHC examination of 80 GC specimens demonstrated significant overexpression of DSCC1 in GC tissues compared to non-cancerous tissues. DSCC1 expression was notably higher in GC tissues than in adjacent non-cancerous tissues **(Figure 2G)**. The relationship between DSCC1 expression and overall survival (OS) in 80 GC patients was evaluated to investigate the connection between DSCC1 expression and GC prognosis. Kaplan-Meier analysis showed that high DSCC1 protein expression was significantly associated with poor OS in GC patients **(Figure 2H)**. Furthermore, statistical analysis of clinicopathological data from all GC patients revealed that high DSCC1 expression was closely linked to tumor size, depth of invasion, lymph nodes metastasis, distant metastases, TNM stage, and degree of differentiation (P < 0.05). However, no significant association was found between DSCC1 expression and other clinicopathological features such as age, gender, lauren type, histological grade, venous invasion, and nerve invasion (P > 0.05) **(Supplementary Table 5)**.

### DSCC1 promotes malignant behavior in GC cells

**Figure 3:**
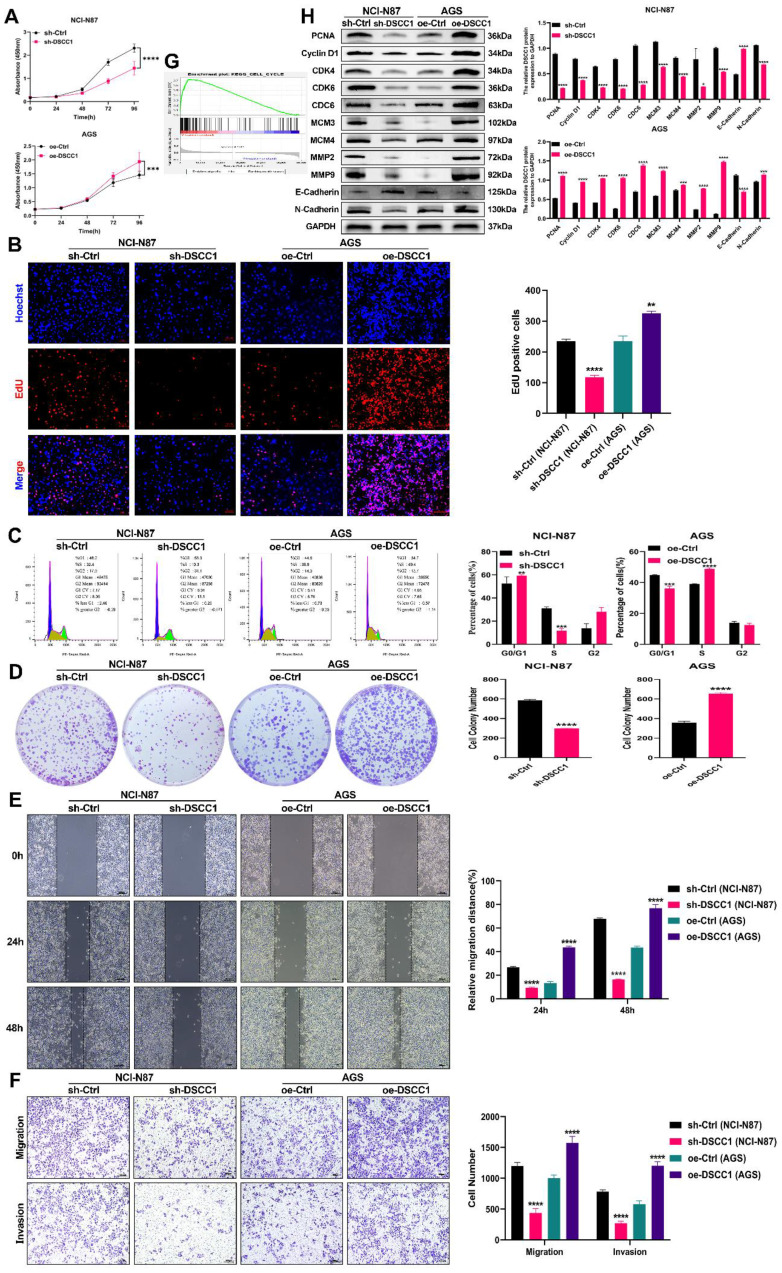
** Role of DSCC1 in Promoting Malignancy in GC Cells. (A-B)** Impact of DSCC1 knockdown or overexpression on GC cell proliferation assessed through CCK-8 assay **(A)** and EdU assay **(B)**. **(C)** Influence of DSCC1 knockdown or overexpression on GC cell cycle analyzed using flow cytometry. **(D)** Effect of DSCC1 knockdown or overexpression on GC cell proliferation measured by colony formation assay. **(E-F)** Impact of DSCC1 knockdown or overexpression on GC cell migration and invasion evaluated through transwell **(E)** and wound healing assay **(F)**. **(G)** GSEA analysis illustrating the correlation between DSCC1 expression and the cell cycle pathway. **(H)** Alterations in cell cycle, proliferation, migration, and invasion-related proteins following DSCC1 knockdown or overexpression analyzed by Western blot in GC cells. (*P<0.05, **P<0.01, ***P<0.001, ****P<0.0001) - Significance levels denoted by asterisks.

To investigate the effects of DSCC1 on the malignant behaviour of GC cells, we measured its expression in normal gastric epithelial cells (GES-1) and various GC cell lines (AGS, NCI-N87, HGC-27, and BGC-823). DSCC1 expression was highest in NCI-N87 cells and lowest in AGS cells **(Figure 2F)**. Based on these findings, we generated stable DSCC1 knockdown and overexpressing cell lines. RT-qPCR and western blot analyses confirmed the successful knockdown and overexpression **(Supplementary Figures 2A-B)**. We then assessed the impact of DSCC1 on GC cell proliferation using CCK-8, EdU, and colony formation assays. The results showed that DSCC1 knockdown inhibited NCI-N87 cell proliferation, whereas DSCC1 overexpression enhanced AGS cell proliferation **(Figure 3A-B, 3D)**. Our primary objective was to understand the role of the DSCC1 gene in the cell cycle, particularly its involvement in the G1/S phase transition. Flow cytometry analysis revealed that DSCC1 knockdown inhibited cell cycle progression in NCI-N87 cells, while its overexpression promoted cell cycle progression in AGS cells **(Figure 3C)**. Meanwhile, the mRNA expression level of DSCC1 in GC was divided into high and low expression groups based on the median value for GSEA enrichment analysis. This analysis revealed that upregulation of DSCC1 could activate the cell cycle signaling pathway **(Figure 3G)**. Additionally, western blot analysis indicated that DSCC1 knockdown reduced the expression of cell cycle and proliferation-related proteins, including PCNA, Cyclin D1, CDK4, CDK6, CDC6, MCM3, and MCM4 in NCI-N87 cells. Conversely, DSCC1 overexpression increased the expression of these proteins in AGS cells **(Figure 3H)**. To examine the relationship between DSCC1 and GC cell migration and invasion, we performed transwell and wound healing assays. DSCC1 knockdown inhibited migration and invasion in NCI-N87 cells, whereas DSCC1 overexpression enhanced these behaviours in AGS cells **(Figure 3D-E)**. Further western blot analysis showed that DSCC1 knockdown decreased the expression of migration and invasion-related proteins MMP2, MMP9, and N-cadherin, while increasing E-cadherin expression in NCI-N87 cells. In contrast, DSCC1 overexpression increased the expression of MMP2, MMP9, and N-cadherin, while decreasing E-cadherin expression in AGS cells **(Figure 3H)**. Overall, DSCC1 was found to promote the proliferation, migration, and invasion of GC cells.

### DSCC1 promotes tumor growth and metastasis *in vivo*

**Figure 4:**
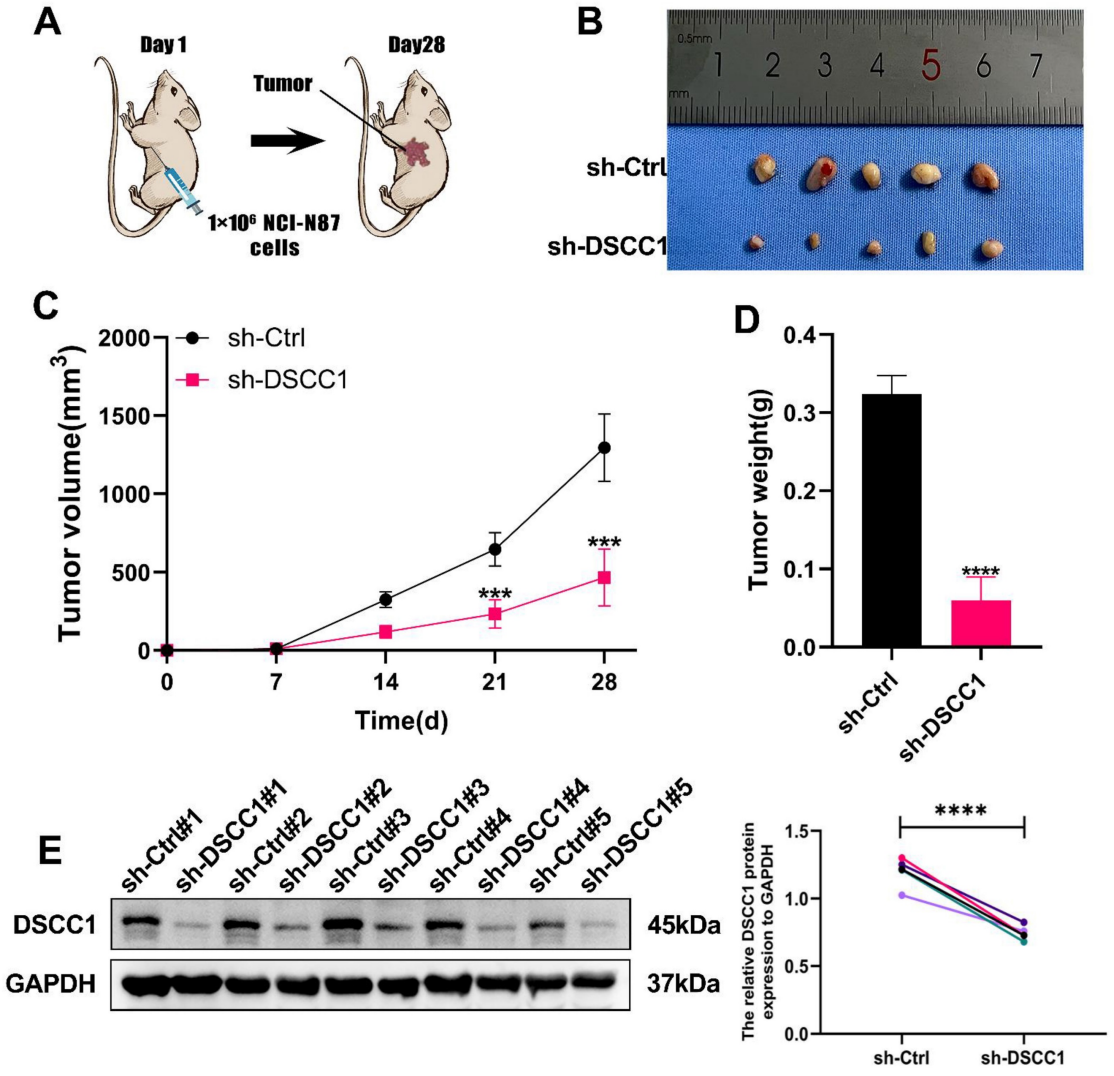
** Role of DSCC1 in Promoting Gastric Cancer Process *In Vivo*. (A)** Inhibition of DSCC1 suppressed the growth of subcutaneous xenograft tumors in nude mice. **(B)** Images of subcutaneous tumors. **(C)** Growth curve of subcutaneous tumors in nude mice. **(D)** Measurement of tumor weight post-sacrifice. **(E)** Western blot analysis of DSCC1 expression in xenograft tumors. (*P<0.05, **P<0.01, ***P<0.001, ****P<0.0001) - Significance levels denoted by asterisks.

To further validate our *in vitro* findings, we investigated the role of DSCC1 in regulating tumor growth and metastasis *in vivo*. We established a subcutaneous tumor model in nude mice by injecting NCI-N87 cells transfected with either sh-Ctrl or sh-DSCC1. We monitored changes in tumor volume and plotted the growth curves accordingly. As anticipated, DSCC1 knockdown significantly slowed tumor growth in the nude mice **(Figure 4A)**. Tumors were harvested, measured for volume, and weighed from the sacrificed animals, revealing that tumors in the sh-Ctrl group were significantly heavier than those in the sh-DSCC1 group **(Figure 4B-D)**. Additionally, Western blot analysis showed that DSCC1 knockdown reduced its expression in the tumor tissues **(Figure 4E)**.

### E2F4-mediated transcriptional upregulation of DSCC1 in GC

**Figure 5:**
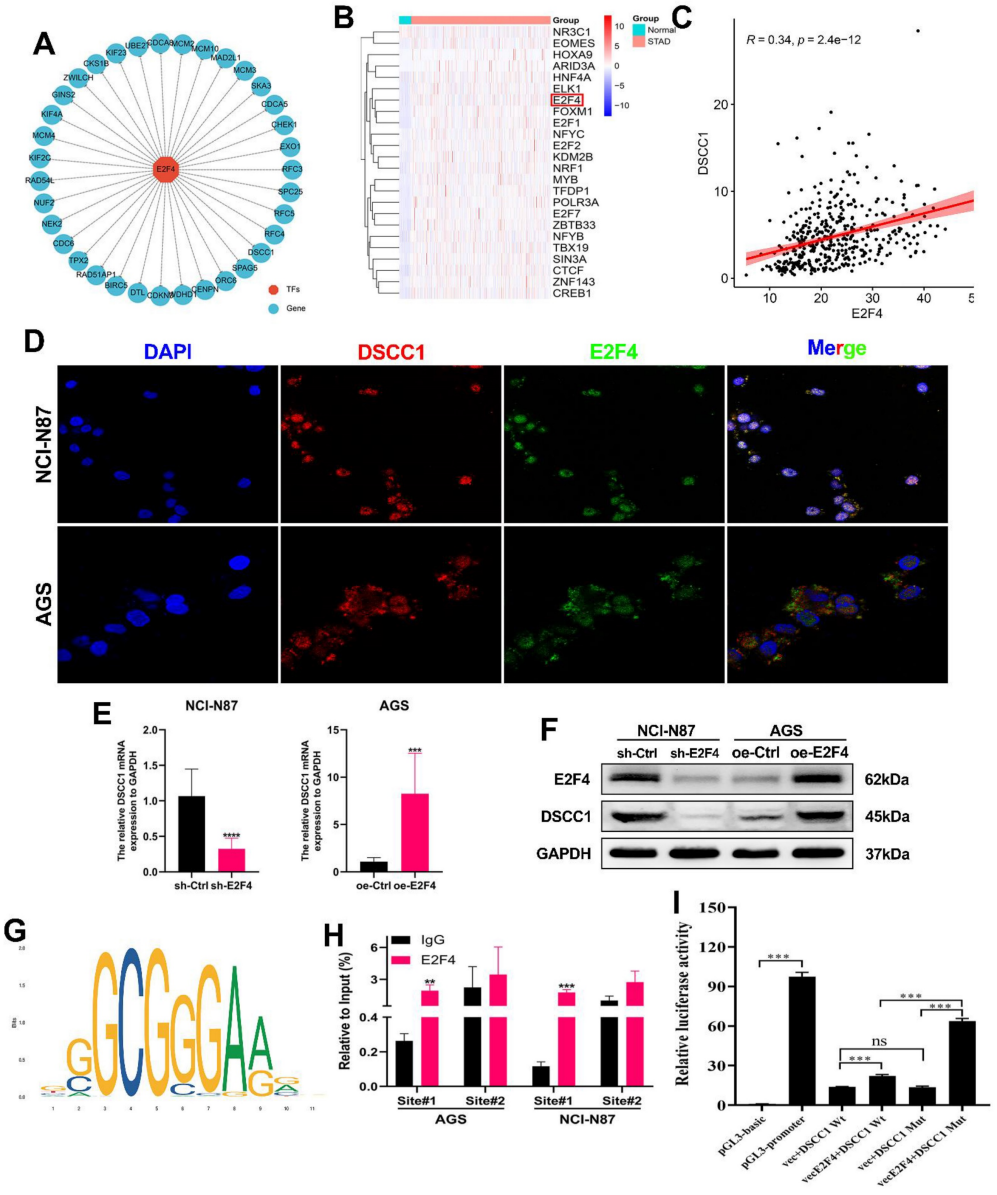
** E2F4 Regulation of DSCC1 Transcription and Expression in GC. (A)** Detection of differentially expressed genes (DEGs) within the major cluster as targets identified by enriched motifs/tracks associated with the E2F4 transcription factor. **(B)** Heatmap illustrating the expression of transcription factors regulating DSCC1 in GC and normal samples. **(C)** Analysis of expression correlations between E2F4 and PLAUR based on TCGA-seq data. **(D)** Representative confocal microscopy image demonstrating the co-localization of E2F4 protein (green) and DSCC1 (red) in AGS and NCI-N87 cell lines. **(E-F)** Positive regulatory effect of E2F4 on DSCC1 expression in GC cells. Evaluation of E2F4 silencing on DSCC1 mRNA and protein expression in GC cells using RT-qPCR **(E)** and Western blot **(F)**, respectively. **(G)** Prediction of the E2F4 binding motif within the DSCC1 promoter through the JASPAR dataset. **(H)** ChIP-qPCR analysis indicating E2F4 binding to the DSCC1 promoter in AGS and NCI-N87 cell lines, with the first position being the most significant binding site. **(I)** Luciferase activity assessment following mutation of the first E2F4 site in the DSCC1 promoter in 293T cell lines. The luciferase activity of the wild-type DSCC1 promoter was notably higher than that of the mutant DSCC1 promoter. (*P<0.05, **P<0.01, ***P<0.001, ****P<0.0001) - Significance levels indicated by asterisks.

As previously mentioned, DSCC1 is upregulated in GC, contributing to tumor malignancy. To investigate the reasons behind DSCC1 upregulation in GC, we first ruled out DSCC1 mutations as a cause using bioinformatics **(Supplementary Figure 3)**. Given the significant role of abnormal transcriptional regulation in cancer development, we hypothesized that aberrant transcription leads to increased DSCC1 expression in GC. Additionally, using the iRegulon package in Cytoscape for transcription factor analysis of the 35 hub genes, we identified 24 highly significant transcription factors **(Supplementary Table 6)**, with E2F4 emerging as the most prominent. E2F4 displayed a normalized enrichment score (NES) of 14.705, originating from the major cluster. The specific tracks associated with E2F4 revealed 27 targets among the 35 DEGs **(Figure 5A)**, and TCGA-STAD-seq data to generate an expression heatmap depicting these transcription factors in GC **(Figure 5B)**. Using the TCGA-STAD-seq data, we examined the relationship between DSCC1 expression and the E2F4 transcription factor in GC **(Figure 5C)**. To explore the regulation of DSCC1 expression by E2F4 at the transcriptional level, we studied the localization of E2F4 and DSCC1 proteins in NCI-N87 and AGS cells using IF staining. E2F4 protein was detected at various levels in the nucleus and cytoplasm of both cell types **(Figures 5D)**. Analysis of fluorescence intensity revealed a positive correlation between E2F4 and DSCC1 expression. Subsequently, a co-immunoprecipitation (co-IP) assay was performed to determine if there was a direct protein-protein interaction between E2F4 and DSCC1, which could regulate abnormal DSCC1 expression in GC. The results indicated no direct interaction between E2F4 and DSCC1 proteins **(Supplementary Figure 4)**. Given that cell nuclei are the primary sites of transcription, it can be deduced that E2F4 regulates DSCC1 expression at the transcriptional level. To validate E2F4's regulatory effect on DSCC1 expression, we performed E2F4 knockdown and overexpression experiments. Knockdown of E2F4 significantly inhibited DSCC1 expression in NCI-N87 cells, while its overexpression increased DSCC1 levels in AGS cells, as confirmed by RT-qPCR and western blot **(Figure 5E-F)**.

This confirmed that E2F4 regulates DSCC1 expression in GC. To determine whether E2F4 directly binds to the DSCC1 promoter, we identified multiple binding sites using the JASPAR database **(Figure 5G; Supplementary Table 7)**. For the top two binding sites identified, we conducted ChIP analysis in NCI-N87 and AGS cells. **Figure 5G** shows that site#1 in the DSCC1 promoter region are primary binding sites for E2F4. To further validate E2F4's transcriptional activation effect on DSCC1, we performed a dual luciferase reporter assay using 293T cells. Based on the ChIP-qPCR results, we mutated the primary E2F4 binding sites in the DSCC1 promoter and examined luciferase activity. The wild-type DSCC1 promoter showed significantly higher luciferase activity than the mutant promoter **(Figure 5I)**. This indicates that the transcription factor E2F4 positively regulates DSCC1 expression. In summary, E2F4 promotes DSCC1 expression through transcriptional upregulation.

### E2F4 expression and its prognostic significance in GC

**Figure 6:**
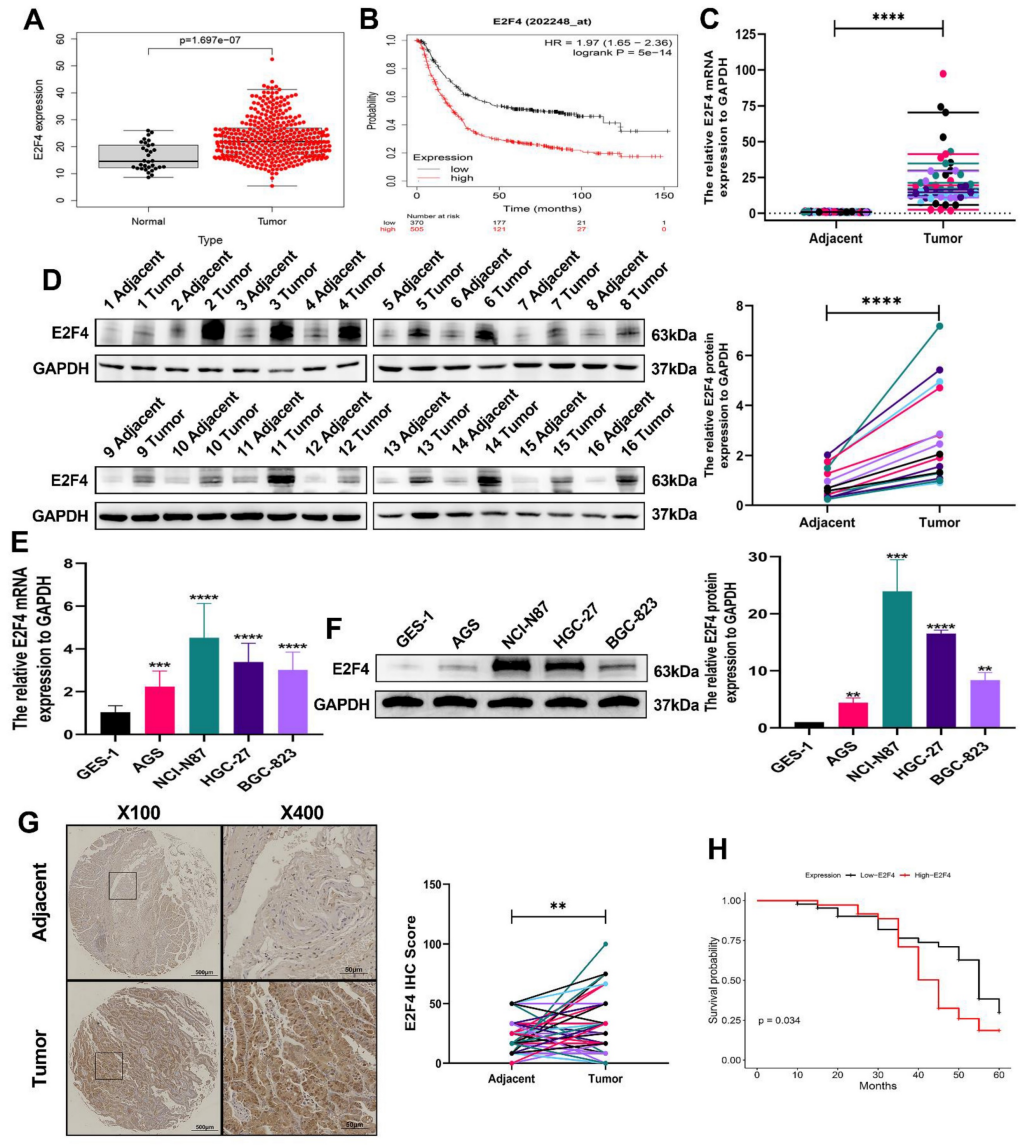
** Up-regulation of E2F4 in GC tissues and cells. (A)** Comparison of F2F4 expression between tumor tissues and adjacent tissues from the TCGA database. **(B)** Comparison of E2F4 expression levels between high and low expression groups from the Kaplan-Meier Plotter. **(C)** Detection of E2F4 mRNA expression in 16 pairs of GC and corresponding adjacent tissues using RT-qPCR. **(D)** Detection of E2F4 protein expression in 16 pairs of GC and corresponding adjacent tissues using Western blot. **(E)** Analysis of E2F4 mRNA and protein expression in GES-1 and GC cell lines by RT-qPCR. **(F)** Analysis of E2F4 mRNA and protein expression in GES-1 and GC cell lines by Western blot.** (G)** Detection of E2F4 expression in 80 paired GC and adjacent non-tumor tissues by IHC. **(H)** Survival analysis of GC patients with varying levels of E2F4 expression. (*P<0.05, **P<0.01, ***P<0.001, ****P<0.0001) - Significance levels denoted by asterisks.

To validate the expression of the E2F4 gene and its relationship with overall survival in STAD patients, we analyzed data from the TCGA database and utilized the Kaplan-Meier plotter. The correlation between E2F4 expression levels and overall survival in STAD patients is shown in **(Figure 6A-B)**. The analysis revealed a significant difference in E2F4 expression levels between STAD samples and normal tissues. Additionally, RT-qPCR and western blot experiments confirmed that E2F4 RNA and protein levels were significantly higher in 16 pairs of fresh GC specimens compared to normal tissues **(Figure 6C-D)**. Further validation through RT-qPCR and western blot analysis of various cell lines showed increased E2F4 expression in four GC cell lines (AGS, NCI-N87, HGC-27, and BGC-823) compared to the normal cell line GES-1 **(Figure 6E-F)**. IHC examination of 80 GC specimens demonstrated significant overexpression of E2F4 in GC tissues relative to non-cancerous tissues, with notably higher E2F4 expression in GC tissues compared to adjacent non-cancerous tissues **(Figure 6G)**. To investigate the statistical analysis of clinicopathological data from all GC patients revealed that high E2F4 expression was closely linked to tumor size, lymph nodes metastasis, distant metastases, and TNM stage, (P < 0.05). However, there was no significant association between E2F4 expression and other clinicopathological features such as age, gender, lauren type, depth of invasion, degree of differentiation, histological grade, venous invasion, and nerve invasion (P > 0.05) **(Supplementary Table 8)**. According to Kaplan-Meier analysis, increased E2F4 protein expression was significantly associated with poor overall survival (OS) in GC patients **(Figure 6H)**. Univariate regression analysis identified several factors affecting OS (P < 0.05): tumor size, lymph nodes metastasis, distant metastases, TNM stage (stages I and II vs. stages III and IV), E2F4 expression (low vs. high), and DSCC1 expression (low vs. high). Multivariate regression analysis confirmed these factors as independent risk factors for GC progression (P < 0.05): tumor size, lymph nodes metastasis, distant metastases, TNM stage, E2F4 expression, and DSCC1 expression **(Supplementary Table 9)**. These results indicate that E2F4 is elevated in GC and that patients with high E2F4 levels have a poor prognosis.

### The role of E2F4 in GC proliferation, migration, and invasion: *in vitro* and *in vivo* studies

**Figure 7:**
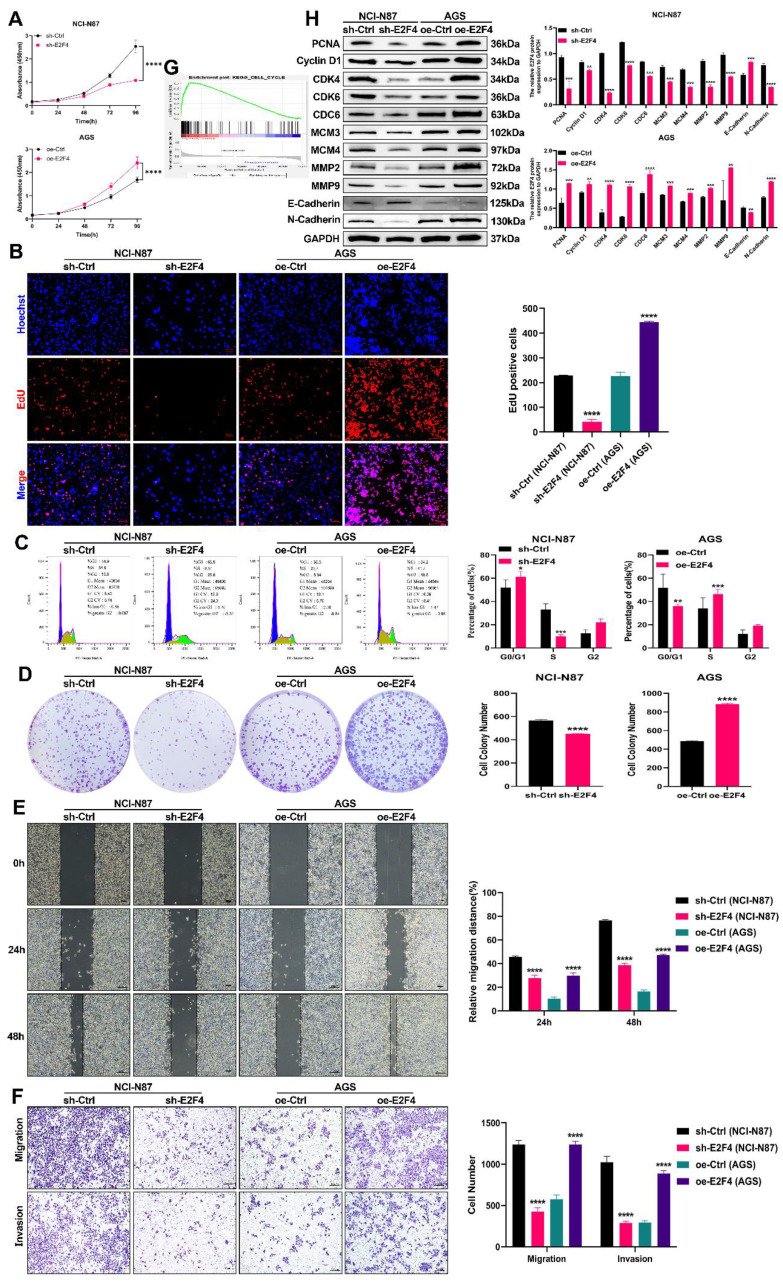
** Role of E2F4 in Promoting Malignancy in GC Cells. (A-B)** Impact of E2F4 knockdown or overexpression on GC cell proliferation assessed through CCK-8 assay **(A)** and EdU assay **(B)**. **(C)** Influence of E2F4 knockdown or overexpression on GC cell cycle analyzed using flow cytometry. **(D)** Effect of E2F4 knockdown or overexpression on GC cell proliferation measured by colony formation assay. **(E-F)** Impact of E2F4 knockdown or overexpression on GC cell migration and invasion evaluated through transwell **(E)** and wound healing assay **(F)**. **(G)** GSEA analysis illustrating the correlation between E2F4 expression and the cell cycle pathway. **(H)** Alterations in cell cycle, proliferation, migration, and invasion-related proteins following E2F4 knockdown or overexpression analyzed by Western blot in GC cells. (*P<0.05, **P<0.01, ***P<0.001, ****P<0.0001) - Significance levels denoted by asterisks.

We generated stable cell lines with E2F4 knockdown and overexpression. The successful manipulation was confirmed via RT-qPCR and Western blot analyses **(Supplementary Figures 2C-D)**. We then investigated E2F4's role in carcinogenesis both *in vitro* and *in vivo*. As shown in **Figures 7A-B and 7D**, E2F4 knockdown inhibited the proliferation of NCI-N87 cells, while E2F4 overexpression promoted proliferation in AGS cells. Flow cytometry analysis revealed that E2F4 knockdown inhibited cell cycle progression in NCI-N87 cells, whereas overexpression enhanced it in AGS cells **(Figure 7C)**. GSEA indicated that high E2F4 expression activated the cell cycle signaling pathway **(Figure 7G)**. Western blot analysis demonstrated that E2F4 knockdown reduced the expression of cell cycle and proliferation-related proteins, including PCNA, Cyclin D1, CDK4, CDK6, CDC6, MCM3, and MCM4 in NCI-N87 cells, while E2F4 overexpression increased these proteins in AGS cells **(Figure 7H)**. Transwell and wound healing assays showed that E2F4 knockdown inhibited migration and invasion in NCI-N87 cells, whereas overexpression enhanced these behaviors in AGS cells **(Figures 7D-E)**. Further western blot analysis indicated that E2F4 knockdown decreased the expression of migration and invasion-related proteins MMP2, MMP9, and N-cadherin, while increasing E-cadherin expression in NCI-N87 cells. Conversely, E2F4 overexpression increased MMP2, MMP9, and N-cadherin expression while decreasing E-cadherin in AGS cells **(Figure 7H)**. Briefly, our data indicate that E2F4 significantly influences the proliferation, migration, and invasion of gastric cancer cells.

**Figure 8:**
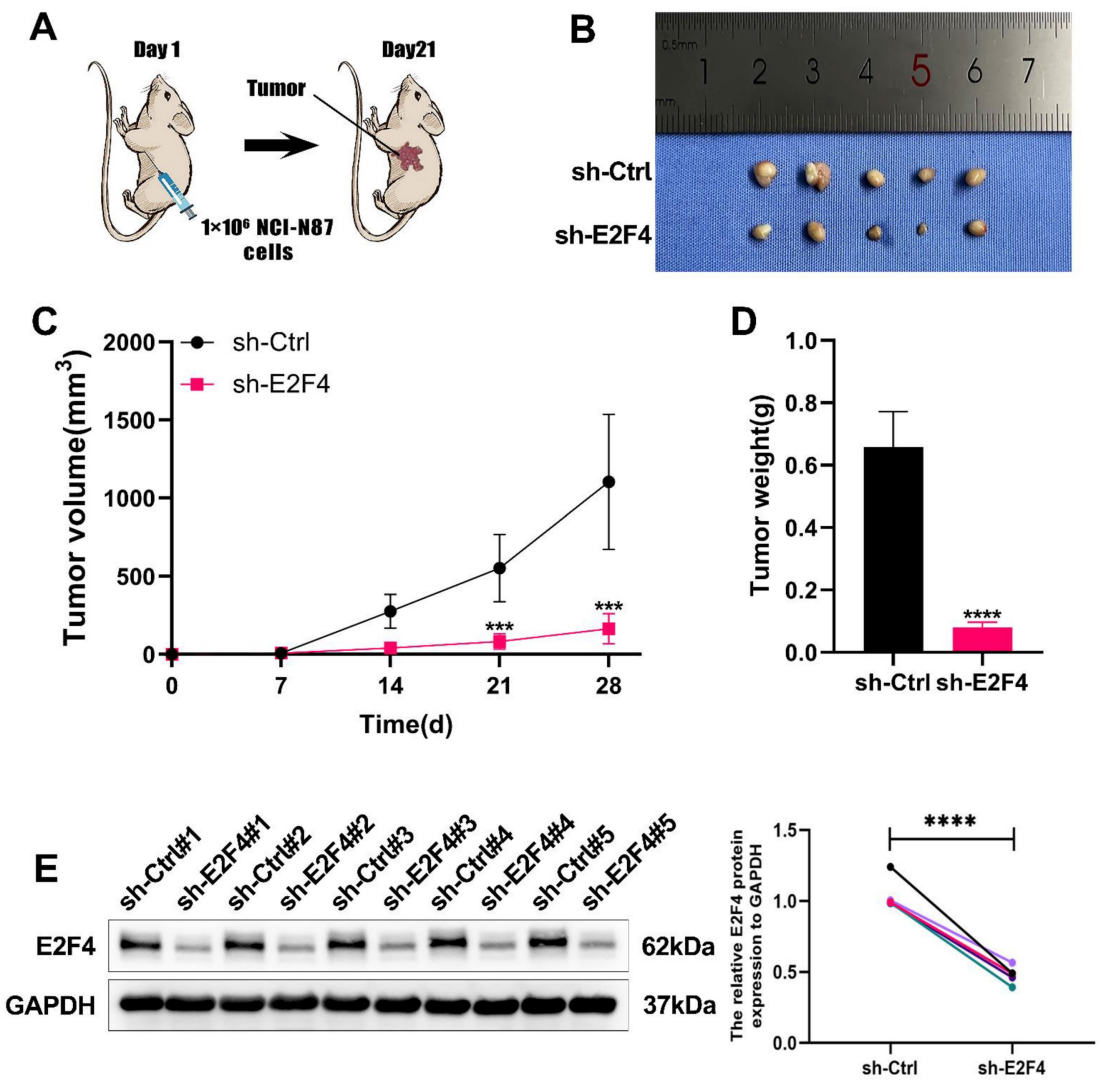
** Role of E2F4 in Promoting Gastric Cancer Process *In Vivo*. (A)** Inhibition of E2F4 suppressed the growth of subcutaneous xenograft tumors in nude mice. **(B)** Images of subcutaneous tumors. **(C)** Growth curve of subcutaneous tumors in nude mice. **(D)** Measurement of tumor weight post-sacrifice. **(E)** Western blot analysis of E2F4 expression in xenograft tumors. (*P<0.05, **P<0.01, ***P<0.001, ****P<0.0001) - Significance levels denoted by asterisks.

To elucidate the functions of E2F4 *in vivo*, we established subcutaneous tumor models in nude mice by injecting NCI-N87 cells, following previously reported methods. The sh-Ctrl group exhibited significantly heavier tumors compared to the sh-E2F4 group **(Figure 8B)**. As shown in **Figure 8C-8D**, measured for volume and tumor weights were measured after euthanizing the mice. Western blot analysis confirmed that E2F4 knockdown effectively reduced its expression in the tumor tissues **(Figure 8E)**. In summary, E2F4 was found to promote the growth and metastasis of GC and regulate the expression of DSCC1 *in vivo*.

### E2F4-DSCC1 axis in regulating proliferation, migration, and invasion of NCI-N87 GC cells

**Figure 9:**
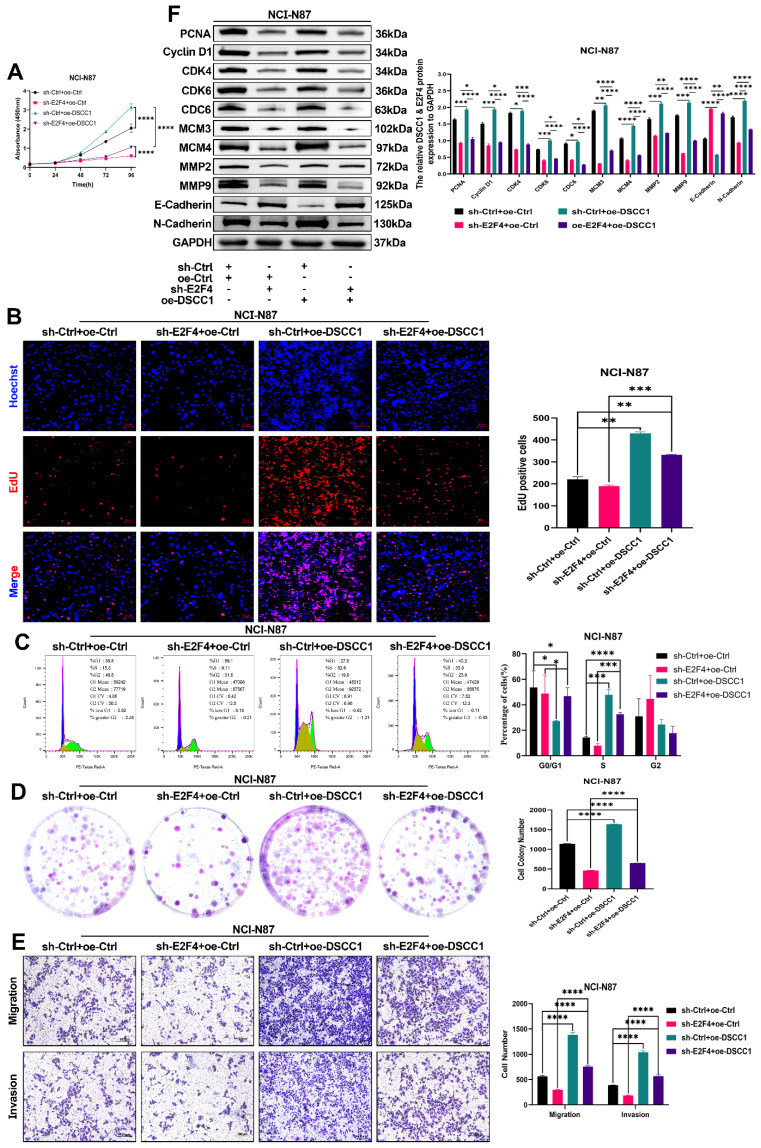
** DSCC1 is Crucial for E2F4-Mediated Malignant Phenotype in GC Cells. (A-B, D)** Cell proliferation in NCI-N87 cells with E2F4 knockdown, control, and DSCC1 re-expression in E2F4-depleted cells, quantified using CCK-8 **(A)**, EdU **(B)**, and colony formation **(D)** assays. **(C)** Cell cycle analysis via flow cytometry in NCI-N87 cells with E2F4 knockdown, control, and DSCC1 re-expression in E2F4-depleted cells. **(E)** Assessment of cell migration and invasion through transwell assays in NCI-N87 cells with E2F4 knockdown, control, and DSCC1 re-expression in E2F4-depleted cells. **(F)** Western blot analysis of cell cycle, proliferation, migration, and invasion-related proteins in NCI-N87 cells with E2F4 knockdown, control, and DSCC1 re-expression in E2F4-depleted cells. (*P<0.05, **P<0.01, ***P<0.001, ****P<0.0001) - Significance levels denoted by asterisks.

To further confirm that DSCC1 is a downstream target gene of E2F4, we conducted rescue experiments in sh-E2F4 NCI-N87 cells by overexpressing DSCC1 and observing changes in proliferation, migration, and invasion. CCK8, EdU, and colony formation assays demonstrated that E2F4 knockdown inhibited NCI-N87 cell proliferation, and this inhibition was reversed by co-transfection with DSCC1 **(Figure 9A-B, D)**. Additionally, as shown in **Figure 9C**, E2F4 knockdown inhibited the cell cycle progression of NCI-N87 cells, which was partially reversed by DSCC1 overexpression. Western blot analysis revealed that the sh-E2F4-induced decrease in the expression of PCNA, Cyclin D1, CDK4, CDK6, CDC6, MCM3, MCM4, MMP2, MMP9, E-Cadherin, and N-Cadherin in suspended NCI-N87 cells was partially reversed by DSCC1 overexpression **(Figure 9F)**. In transwell assays, E2F4 knockdown inhibited the migration and invasion abilities of NCI-N87 cells, and this reduction was partially rescued by co-transfection with DSCC1 **(Figure 9E)**. These results indicate that E2F4 promotes the proliferation, migration, and invasion of GC cells through a DSCC1-dependent mechanism.

### Combination of E2F4 inhibitor and the cell cycle pathway inhibition for the therapy of GC

**Figure 10:**
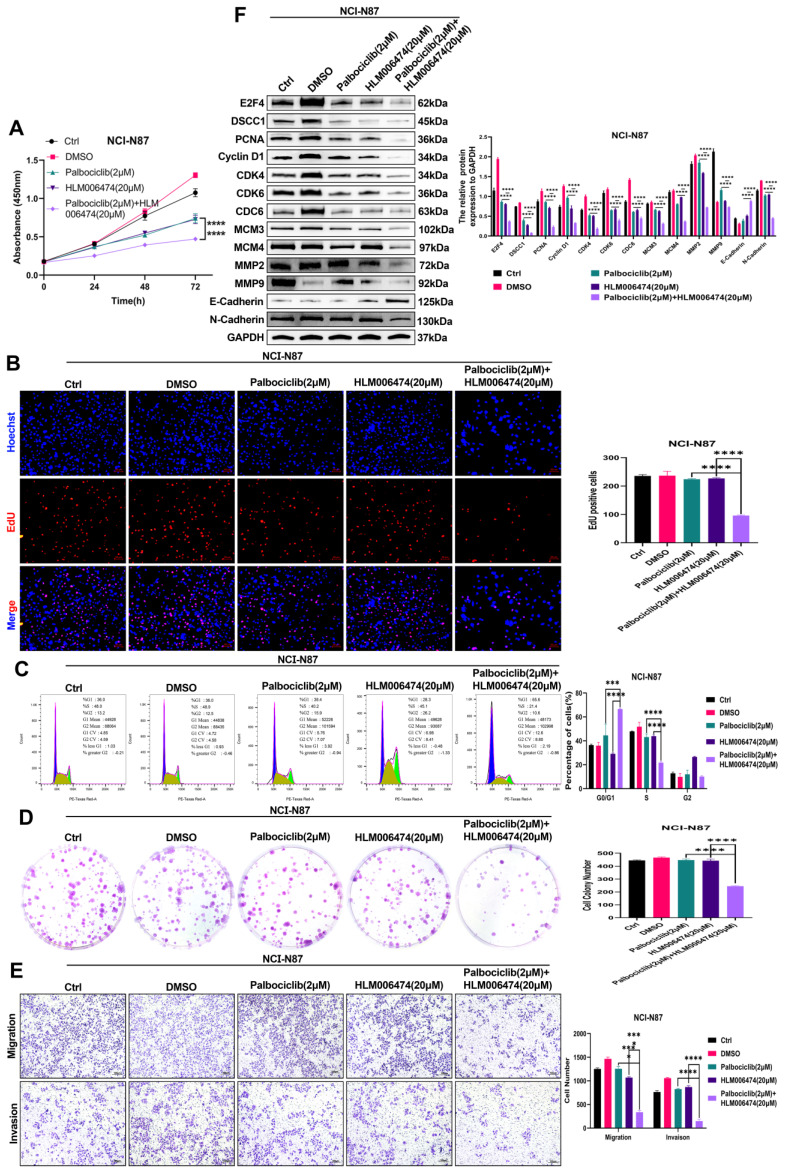
** (A-F) The effect of combination HLM006474 and Palbociclib on the Proliferation, Migration, Invasion and cell cycle in NCI-N87 cells.** CCK8 assay **(A)**, proliferation measured by EdU assay **(B)**, cell cycle progression analysis **(C)**, colony formation ability **(D)**, migration and invasion capabilities determined through transwell assays **(E)**, and Western blot analysis of proteins related to cell cycle, proliferation, migration, and invasion **(F)** in NCI-N87 cells treated with the combination of HLM006474 and Palbociclib. (*P<0.05, **P<0.01, ***P<0.001, ****P<0.0001) - Significance levels denoted by asterisks.

To assess the impact of cell cycle inhibitors on NCI-N87 cell viability, we treated the cells with varying concentrations of Palbociclib (1, 2, 4, 6, 8, and 10 μM) for 24, 48, and 72 hours, and E2F4 inhibitor HLM006474 (10, 20, 40, 60, 80, and 100 μM) for the same time periods **(Supplementary Figures 5A-B)**. To investigate the resistance mechanisms of NCI-N87 cells to HLM006474, we analyzed the expression and activation of related proteins. Treatment with HLM006474 at 80 and 100 μM significantly reduced the activation of CDK4 and CDK6 **(Supplementary Figures 5C)**. Compared to control groups, treatment with HLM006474 (2 μM) or Palbociclib (20 μM) alone had no effect on cell viability. However, the combination of HLM006474 (2 μM) and Palbociclib (20 μM) significantly inhibited cell viability compared to either HLM006474 or Palbociclib alone **(Figure 10A-B, D-E)**. Compared to control groups, HLM006474 (2 μM) and Palbociclib (20 μM) alone had no significant effect on cell cycle progression. However, the combination of HLM006474 (2 μM) and Palbociclib (20 μM) significantly inhibited cell cycle progression compared to either treatment alone. These findings suggest that Palbociclib synergistically enhances the effects of HLM006474 on NCI-N87 cell cycle progression **(Figure 10C)**.

Additionally, we examined the impact of the combination treatment on related proteins. HLM006474 (2 μM) and Palbociclib (20 μM) alone inhibited E-Cadherin activation and upregulated E2F4, DSCC1, PCNA, Cyclin D1, CDK4, CDK6, CDC6, MCM3, MCM4, MMP2, MMP9, and N-Cadherin. In contrast, the combination treatment significantly inhibited the expression of E2F4, DSCC1, PCNA, Cyclin D1, CDK4, CDK6, CDC6, MCM3, MCM4, MMP2, MMP9, and N-Cadherin **(Figure 10F)**. These results indicate that HLM006474 inhibits E-Cadherin activation while promoting E2F4, DSCC1, and other proliferation and migration-related proteins. Palbociclib, as a cell cycle pathway inhibitor, combined with HLM006474, effectively blocks the activation of both pathways.

**Figure 11:**
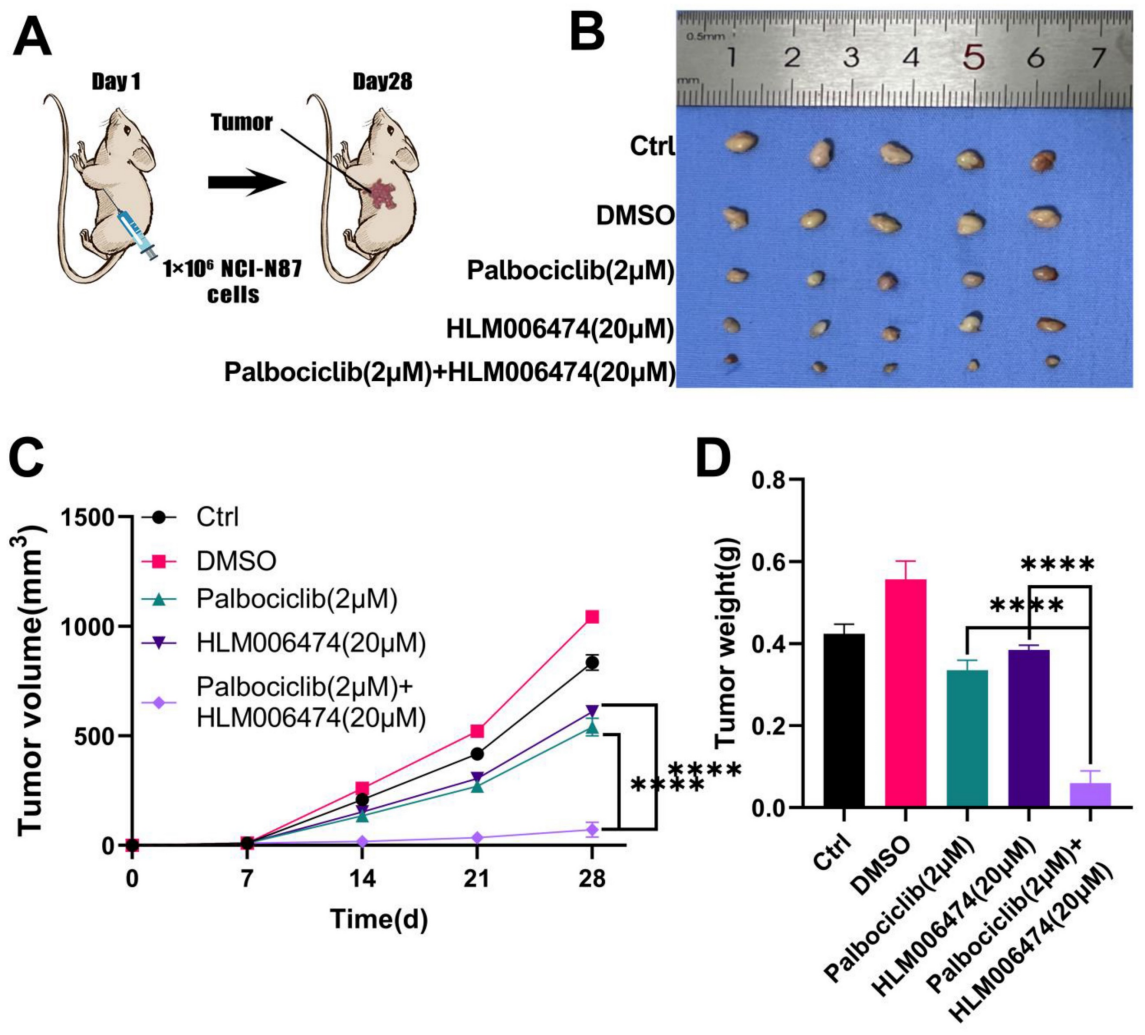
** The effects of combination treatment of HLM006474 and Palbociclib *in vivo*. (A)** Combination treatment of HLM006474 and Palbociclib suppressed the growth of subcutaneous xenograft tumors in nude mice. **(B)** Images of subcutaneous tumors. **(C)** Growth curve of subcutaneous tumors in nude mice. **(D)** Measurement of tumor weight post-sacrifice. (*P<0.05, **P<0.01, ***P<0.001, ****P<0.0001) - Significance levels denoted by asterisks.

To evaluate the effects of combined HLM006474 and Palbociclib treatment *in vivo*, tumors were harvested and examined after 28 days **(Figure 11A-B)**. Tumor volume and weight were measured in NCI-N87 GC cells implanted in nude mice **(Figure 11C-D)**. Starting from day 14, NCI-N87 cells developed large tumors in the mice. HLM006474 and Palbociclib individually did not significantly inhibit tumor growth compared to the control group (no treatment). However, the combination of HLM006474 and Palbociclib showed a significantly stronger inhibitory effect on both tumor volume and weight than either treatment alone.

## Discussion

This research uncovered that E2F4 exhibits high expression levels in GC, correlating closely with unfavorable clinicopathological characteristics in patients. Elevated E2F4 expression independently predicts a poor prognosis for GC patients. *In vitro* experiments confirmed E2F4's role in promoting GC cell proliferation, migration, and invasion. *In vivo* studies further demonstrated that E2F4 enhances tumor growth and metastasis in nude mice. Notably, E2F4 acts as a key transcriptional regulator of DSCC1, activating it by binding to specific sites within the DSCC1 promoter region. Similarly, DSCC1 was found to promote malignancy in GC, particularly cell proliferation, in both *in vitro* and *in vivo* settings. Together, these findings underscore the dysregulated interplay between E2F4 and DSCC1 in GC, contributing significantly to GC cell proliferation, migration, and invasion.

Members of the fibrinolytic system, including DSCC1, play essential roles in cellular processes such as chemotaxis, invasion, migration, regulation of cytokines and growth factors, immune response, and angiogenesis^33^-^35^. DSCC1 specifically contributes significantly to the malignant advancement of GC, particularly in the migration of GC cells. The collagen fibrin network, established in the intercellular space through stromal cell secretion, serves as a barrier against tumor cell migration and invasion. However, tumor cells disrupt this network by activating the fibrinolytic system via DSCC1, thereby facilitating metastasis.^13^, ^36^, ^37^. Our study validated the role of DSCC1 in promoting GC malignancy both *in vitro* and *in vivo*. Additionally, Jin *et al*.^36^ demonstrated elevated DSCC1 expression in breast carcinoma tissues through semi-quantitative IHC. Through Cox analysis, they noted a significant negative correlation between DSCC1 expression and the survival duration of GC patients. Several other studies have also confirmed the high expression of DSCC1 in various cancers, suggesting its significant involvement in tumor distant metastasis^17^, ^38^-^40^. Therefore, DSCC1 emerges as a crucial regulator of cancer cell proliferation and migration. Interestingly, previous studies have not explored the correlation between DSCC1 expression and the proliferation, migration, and invasion of GC cells.

We delved into the molecular mechanism behind DSCC1 upregulation in GC, pinpointing E2F4 as a potential transcriptional regulator. E2F4 protein predominantly localized in the nuclei of GC cells. Through ChIP-qPCR and dual luciferase reporter assays, we confirmed binding sites between E2F4 and the DSCC1 promoter region, validating E2F4's positive regulation of DSCC1 expression in GC. DSCC1's regulation involves various transcription factors; for instance, in colorectal cancer, the upregulated transcription factor E2F1 possesses binding sites within the DSCC1 promoter, resulting in DSCC1 upregulation and impacting colorectal cancer cells^41^. However, E2F4's role in GC remains uncertain, particularly regarding its involvement in GC cell proliferation, migration, and invasion.

E2F4 is a key player in cell cycle progression and cell proliferation. When activated, E2F4 promotes the transcription of genes necessary for transitioning from the G1 phase to the S phase, including those involved in DNA replication and cell division. Conversely, E2F4 can also repress genes related to cell cycle arrest and differentiation^42^-^44^. In head and neck squamous cell carcinoma, high E2F4 expression is linked to poor prognosis, with IHC analysis showing elevated levels of E2F4^45^. However, previous studies have not examined whether E2F4 binds to promoter regions to facilitate cancer cell growth and migration. Our study found that E2F4 is upregulated in GC tissues and cells. *In vitro* experiments demonstrated that E2F4 enhances GC cell proliferation, migration, and invasion. *In vivo* experiments further confirmed that E2F4 promotes tumor growth and metastasis in nude mice. Functional rescue experiments verified that E2F4 significantly contributes to the malignancy of GC in a DSCC1 dependent manner.

Cell division relies on the cell cycle, which is regulated by cyclin-dependent kinases (CDKs). Cyclin D's interaction with CDK4 and CDK6 promotes the phosphorylation of the retinoblastoma protein, advancing the cell cycle from the G1 phase to the S phase^46^. Targeting CDK6 and the closely related CDK4 kinase has garnered significant interest for cancer therapy. Previous research has shown that Palbociclib, a highly selective CDK4/6 inhibitor, can block the G1 to S phase transition in the cell cycle^47^. HLM006474 is predicted to form hydrogen bonds with three conserved residues within the E2F family, suggesting that it is not specific to E2F4 heterodimers^48^. Many E2F-regulated promoters are repressed by E2F4/Rb complexes during G0/G1 and are de-repressed at the G1/S boundary. Blocking E2F DNA-binding activity is predicted to upregulate these genes, potentially increasing cell growth^49^. Our results showed that low concentrations of HLM006474 did not significantly inhibit cell viability, thus there has been no advocacy for HLM006474 as a monotherapy. Abnormalities in the cell cycle pathway contribute to the pathogenesis of several cancers^50^. In this study, we found that HLM006474 significantly inhibited the cell cycle pathway. Consequently, we used the cell cycle inhibitor Palbociclib to observe its combined effect with HLM006474. While HLM006474 alone and Palbociclib alone did not affect cell viability, their combination significantly inhibited cell viability compared to HLM006474 and Palbociclib, respectively.

## Conclusions

In conclusion, E2F4 is highly expressed in GC and serves as an independent predictor of poor prognosis in GC patients. E2F4 promotes the proliferation, migration, and invasion of GC cells through its regulation of DSCC1. Our study elucidates the role of DSCC1 in the upstream molecular regulation of GC, offering a theoretical foundation for targeting E2F4 and DSCC1 as potential therapeutic strategies for GC. Additionally, Combining the E2F4 inhibitor HLM006474 with the cell cycle inhibitor Palbociclib significantly reduced cell viability and inhibited cell cycle progression more effectively than HLM006474 alone. Palbociclib synergistically enhanced the effects of HLM006474 on GC *in vitro* and *in vivo*, supporting the strategy of combining these inhibitors to reduce drug resistance.

## Supplementary Material

Supplementary figures and tables.

## Figures and Tables

**Figure 12:**
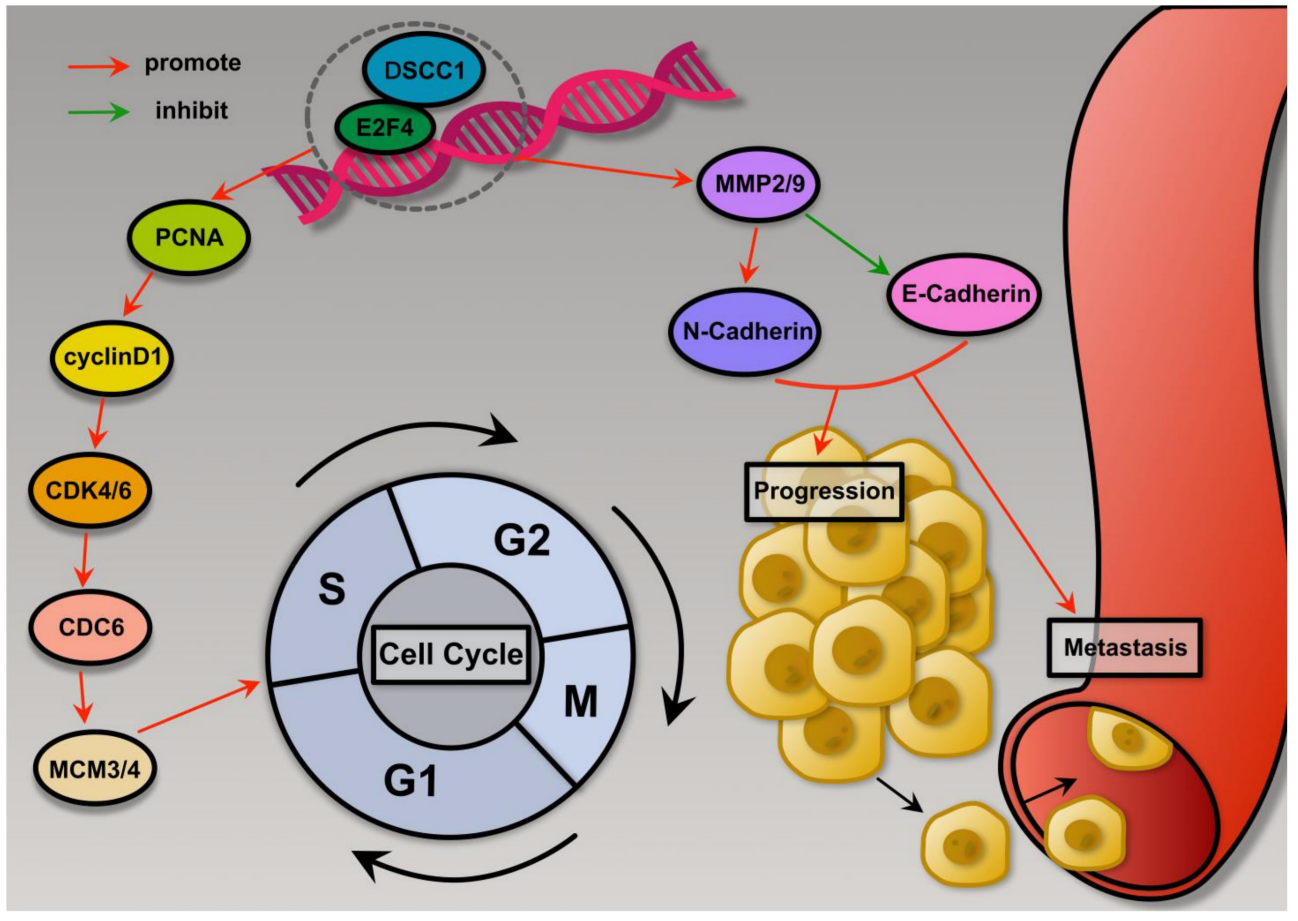
** Schematic summary.** Mechanistically, E2F4 is upregulated in GC and transcriptionally activates DSCC1, which promotes Proliferation, Migration, and Invasion of GC cells.

## Abbreviations

GC: Gastric Cancer
EMT: Epithelial-Mesenchymal Transition
ECM: Extracellular Matrix
DSCC1: DNA Replication and Sister Chromatid Cohesion 1
E2F4: Early 2 Factor 4
ChIP: Chromatin-immunoprecipitation
co-IP: Co-immunoprecipitation
shRNA: small hairpin RNA
IHC: immunohistochemistry
IF: immunofluorescence
STAD: stomach adenocarcinoma
