# Supplementary material for: Transcription Factor E2F4 Promote Proliferation, Migration, and Invasion of Gastric Cancer Cells by transcriptionally activating DSCC1
Source: Int J Biol Sci. 2024 Sep 16;20(12):4978–98. doi: 10.7150/ijbs.99590 (PMC11414385; doi:10.7150/ijbs.99590)
Supplement: Supplementary file 1 — Supplementary figures and tables. [file ijbsv20p4978s1.pdf]

Supplementary materials

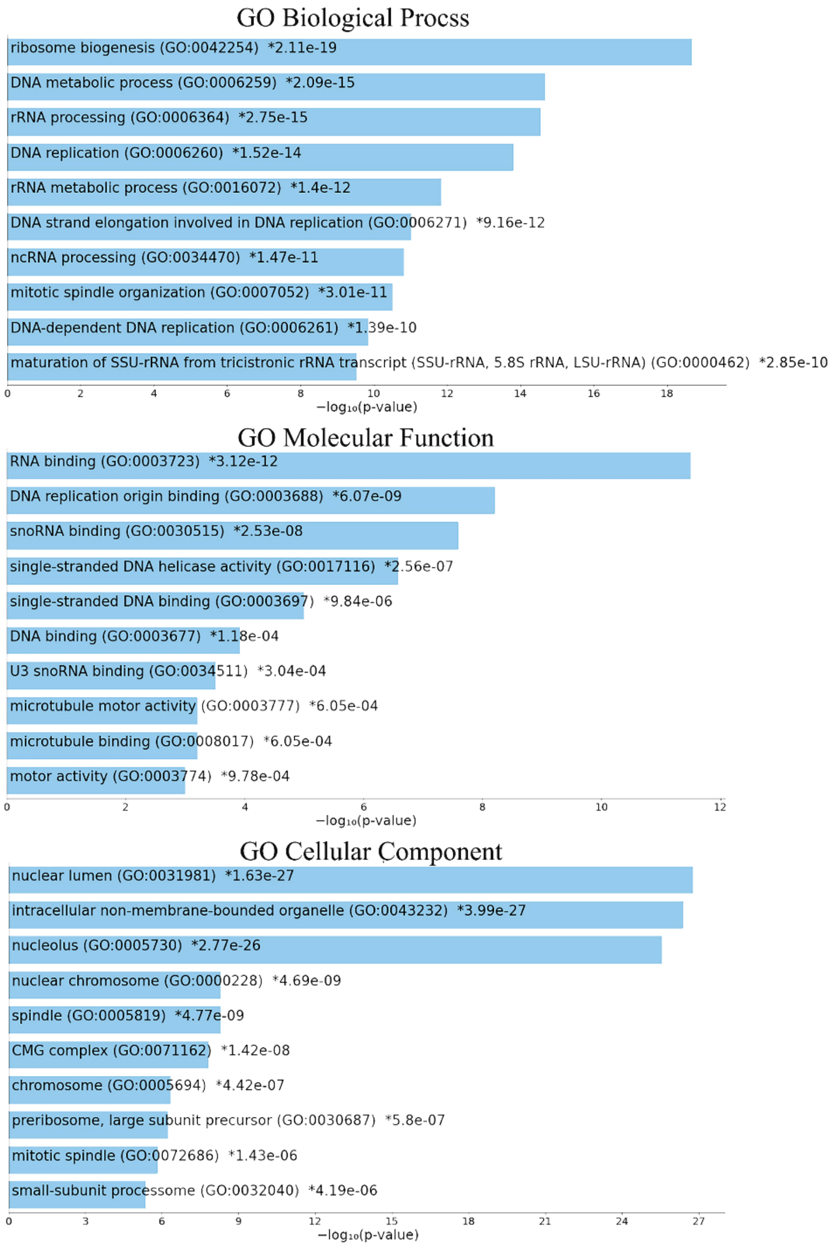

Supplementary Figures 1. Bar chart of the top enriched terms from GO categories: Biological Process, Molecular Function, and Cellular Component. The top 10 enriched terms for the hub genes are shown, ranked by -log10 (p-value), with the actual p-value listed next to each term. The highest-ranked term has the greatest number of hub genes contributing to the GO analysis.

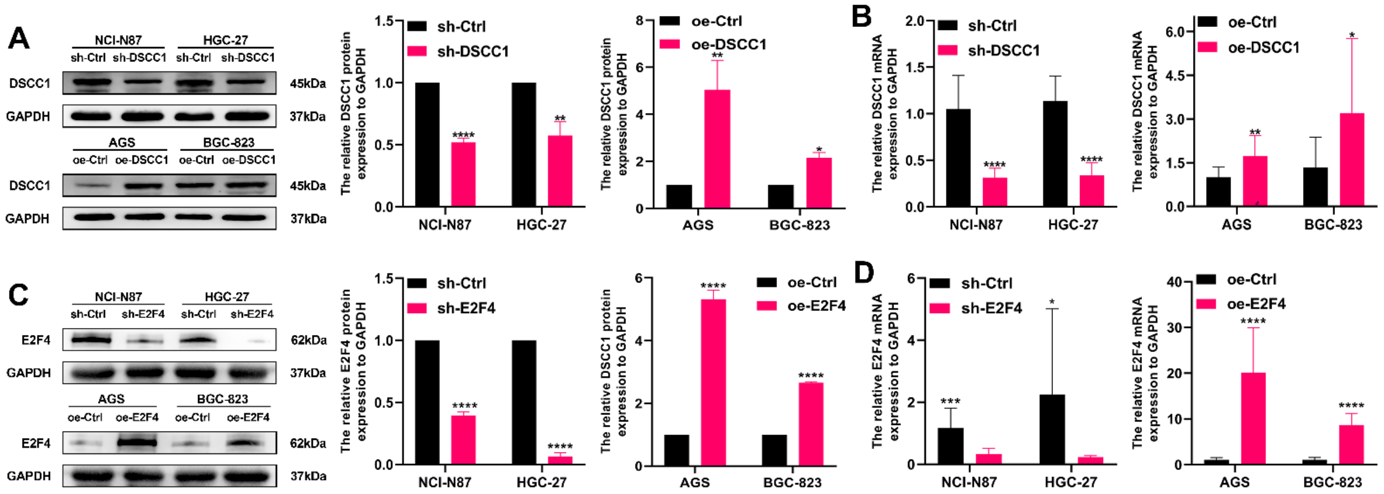

Supplementary Figures 2. Confirmation of DSCC1 inhibition in NCI-N87 and HGC-27 cells and its overexpression in AGS and BGC-823 cells through Western blot **(A)** and RT-qPCR **(B)** analyses, respectively. Confirmation of E2F4 inhibition in NCI-N87 and HGC-27 cells and its overexpression in AGS and BGC-823 cells through Western blot **(C)** and RT-qPCR **(D)** analyses, respectively. (*P<0.05, **P<0.01, ***P<0.001, ****P<0.0001) - Significance levels denoted by asterisks.

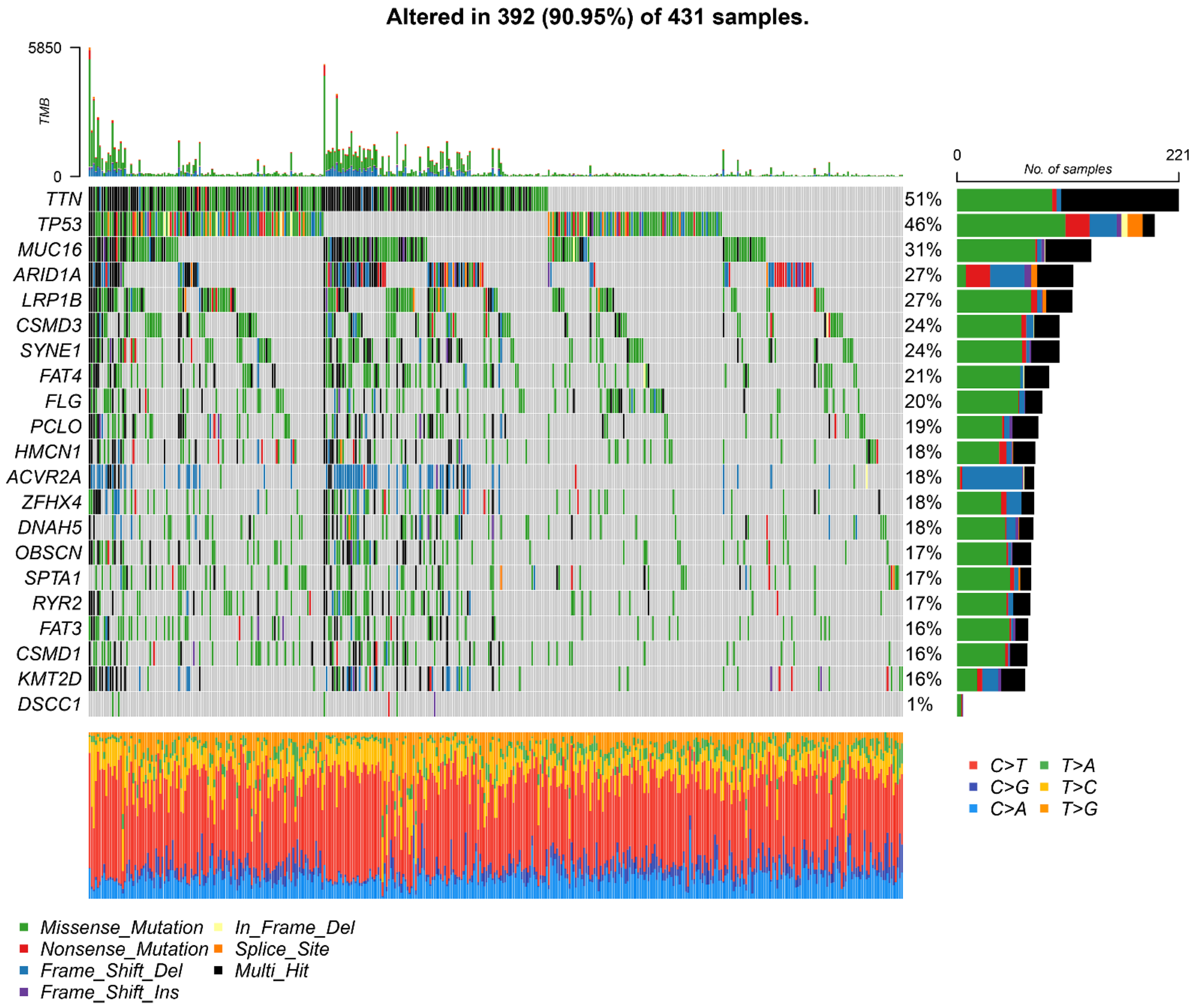

Supplementary Figures 3. Waterfall plot displaying mutated genes in STAD derived from TCGA-STAD sequencing data.

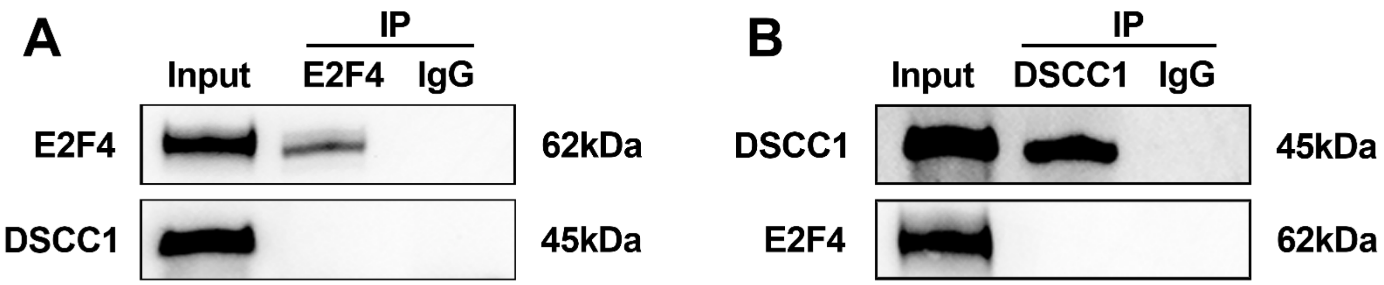

Supplementary Figures 4. The Co-IP analysis revealed the absence of interaction between the E2F4 and DSCC1 proteins.

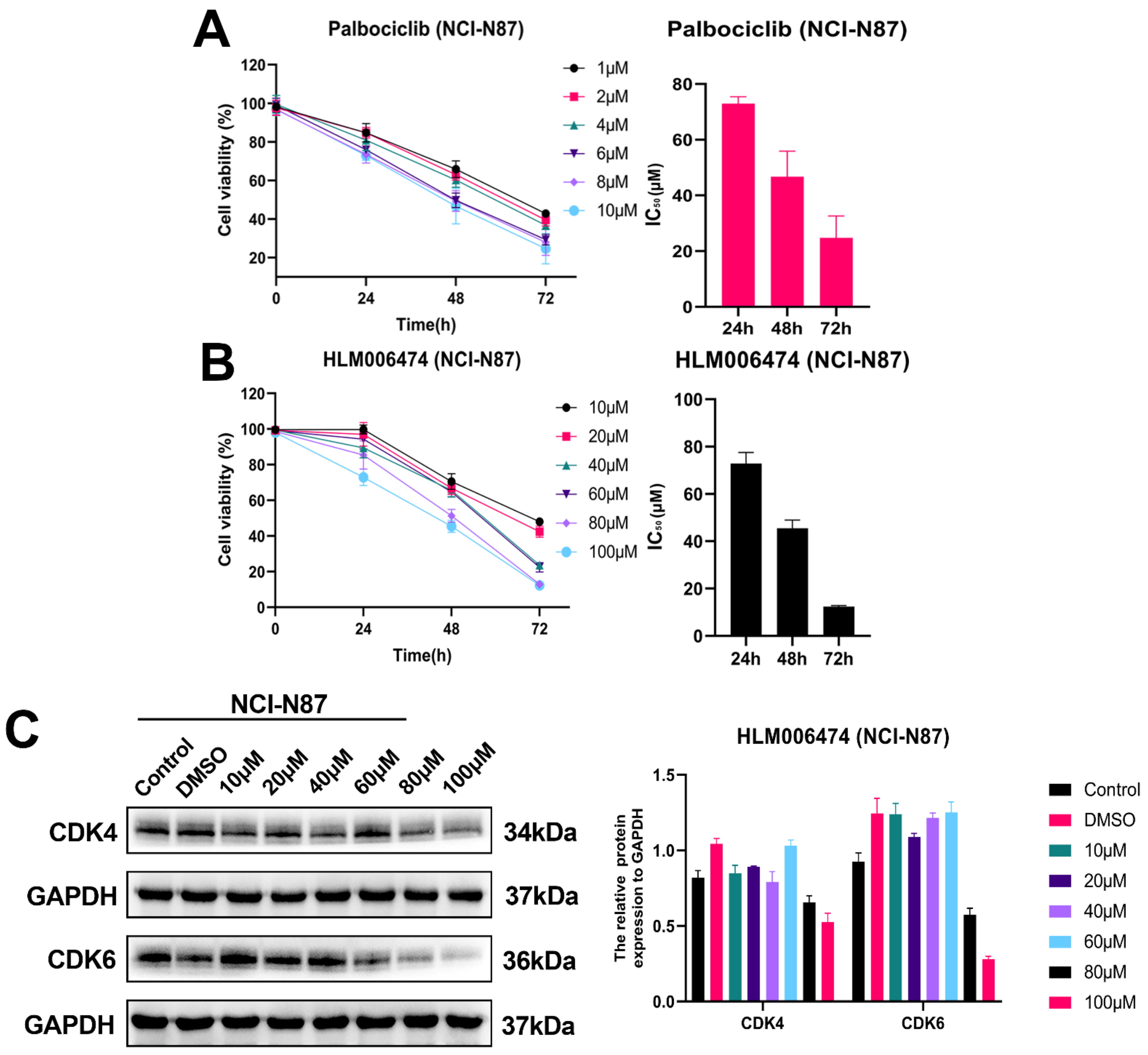

Supplementary Figures 5. (A) The effect of cell cycle inhibitors Palbociclib on NCI-N87 cell viability. **(B)** The effect of E2F4 inhibitors HLM006474 on NCI-N87 cell viability. **(C)** The effect of HLM006474 on CDK4 and CDK6 by Western blot.

Supplementary Table 1. Primer information for RT-qPCR.

Gene Symbol	Forward Primer	Reverse Primer
DSCC1	TCGTGGTGATAAAGACGAGCA	CCGGAGTTTTACAACCAGGAAT
E2F4	CACCACCAAGTTCGTGTCCC	GCGTACAGCTAGGGTGTCA
GAPDH	GGAGCGAGATCCCTCCAAAAT	GGCTGTTGTCATACTTCTCATGG

Supplementary Table 2. The information of primary antibodies.

Antibody name	Manufacturer	Article Number
Anti-DSCC1	Abmart	PHU7133
Anti-DSCC1(IF)	Bioss	bs-7720R
Anti-E2F4	Cell Signaling Technology	#40291
Anti-E2F4(IF)	Bioss	Bs-1399R
Anti-PCNA	Proteintech	10205-2-AP
Anti-Cyclin D1	Proteintech	26939-1-AP
Anti-CDK4	Proteintech	11026-1-AP
Anti-CDK6	Proteintech	14052-1-AP
Anti-CDC6	Proteintech	11640-1-AP
Anti-MCM3	Proteintech	15597-1-AP
Anti-MCM4	Proteintech	13043-1-AP
Anti-MMP2	Proteintech	10373-2-AP
Anti-MMP9	Proteintech	10375-2-AP
Anti-E-cadherin	Proteintech	20874-1-AP
Anti-N-cadherin	Proteintech	22018-1-AP
GAPDH	Proteintech	60004-1-Ig

Supplementary Table 3. The information of primer sequences

Sequence Name	Forward Primer	Reverse Primer
Site#1	GAAGCCGCGTTTCCTTCCTG	GGAAGGTAGGAAACCTGAGCG
Site#2	AGGTCCAGCTCCCTGACAGA	GCTGCGAATCCCAACTCTCT

Supplementary Table 4. Top 5 significant p-values and q-values for KEGG pathways.

KEGG Pathways	p-value	q-value	Genes
DNA replication	9.23E-10	1.18E-08	RFC3, PCNA, RFC4, MCM3, MCM4, MCM2
Ribosome biogenesis in eukaryotes	9.81E-10	1.18E-08	RBM28, LSG1, UTP6, BMS1, HEATR1, NAT10, GNL2, GTPBP4
Cell cycle	7.75E-08	6.20E-07	PCNA, CHEK1, MCM3, MCM4, CDC6, MCM2, MAD2L1
Mismatch repair	5.80E-07	3.48E-06	RFC3, PCNA, RFC4, EXO1
Nucleotide excision repair	0.000360532	0.001730555	RFC3, PCNA, RFC4

Supplementary Table 5. Associations between DSCC1 expression and clinicopathological features of 80 patients with gastric cancer.

Histopathological parameters	Total number (80)	Expression level of DSCC1		P-value
		Low	High	
Age, years				0.547
<65	33	17	16	
≥65	47	21	26	
Gender				0.408
Male	51	26	25	
Female	29	12	17	
Tumor size				0.027
<6	43	26	17	
≥6	37	12	25	
Lauren type				0.593
Intestinal type	73	34	39	
Diffuse type	7	4	3	
Depth of invasion				0.049
T1, T2	29	18	11	
T3, T4	51	20	31	
Lymph nodes metastasis				0.007
N0, N1	40	25	15	
N2, N3	40	13	27	
Distant metastases				<0.001
Negative	58	35	23	
Positive	22	3	19	
TNM stage				0.020
I	12	10	2	
II	17	8	9	
III	39	13	26	
IV	12	7	5	
Degree of differentiation				0.004
Highly	43	14	29	
Moderately and poorly	37	24	13	
Histological grade				0.617
I	8	4	4	
II	38	20	18	
III	34	14	20	
Venous invasion				0.463
No	45	23	22	
Yes	35	15	20	
Nerve invasion				0.102
No	35	13	22	
Yes	45	25	20	

Supplementary Table 6. 24 highly significant transcription factors.

Transcription Factors	NES	Targets
E2F4	14.705	27
TFDP1	11.371	27
NFYC	8.400	20
SIN3A	8.325	33
E2F7	7.160	20
ZNF143	6.452	13
ZBTB33	5.801	16
NFYB	5.728	18
FOXM1	5.482	22
NRF1	5.133	18
NR3C1	4.379	6
CTCF	4.330	14
ELK1	4.261	6
POLR3A	4.192	9
E2F1	4.105	25
MYB	3.935	5
CREB1	3.593	9
ARID3A	3.589	2
KDM2B	3.343	2
HOXA9	3.326	2
E2F2	3.259	26
EOMES	3.208	2
HNF4A	3.178	2
TBX19	3.112	4

Supplementary Table 7. The information of DSCC1 promoter sequence

**DSCC1 promoter sequence**
TGTGCTCTTTATTGAATAAAAAAGAAGAGAAGAGAGGAAAAGAAAAGGAAAAAAGTTCATACATGATTCTATAATATGAACTAAGATTTAGTAAA<br>TAAAATTCTCATTTCATGCCATGAAACACAATATATGAGCAATATAATCAAGAGTACAGTGTCCAAGAGAACAGAATCAATTTTAGCTGATTCTGTT<br>CTCATGAACAGAACTTTTGAAATTTATTATAACTGTTCATTTTTTTACCTGATAAAGAATATTTTTTTCCTGCTACCAGATGAATGATTAAGCCAGCT<br>TCAATCGTTTTTTGAAAAGGTACAGAAAATTTCTTACTTTAGAAAAACATATTAACATAATTTTAAAGAATGAATTAATAAAAACTATCATCATTTT<br>GTCTTATGAGAGGAACACAGGTCCAGCTCCCTGACAGAATATCAAATTAATGGCAATAATTTCATACCATTAATTGGAAATTAAATTCATAATGATT<br>AATACATAAAAACAAAAAGAACTGCTGCCATTTATTAAGCAGCCATGCATTTTAGAATCCTTCCCGCGGGTACATCTTAAGAATGTAGGTCTTATGT<br>GCTTTTTTATCGATAAGGAAGTTGTACTCAGAGAAATTAGGTAAATTGCCTTAGGTCACAAAGTTCTGAAGAGAGTTGGGATTCGCAGCCATGCC<br>TGTCTGATGTAAATGTCAGGGTTCTAGTTGACACTGCTTCTAGTTGTAGGGAATGTTTCCCATTTAATAGAGCAACTACAATGGTTGATTCCCTTCA<br>ATAATAACTTCTTATCAGGCAACCACTGTCACATTCAGGATTTCATTCCACCGAATTCCTATGCATTCAAATGTAAAAGAGGCTAGTGAAGAAGTA<br>GGTCACATGAACCAATCTTTCCACCACCAACGAAAGCACTGCCCAGACTCCTGGGATGCCCTACTCTGGTATTTTCGTATCATTTTACCCAAGAAT<br>TGAAAAAAATGTCTGCTCAGATCCTTTGAATTCAACACCTTACCCACCACTTTCTTTCACTTCAAGCAAGGTCTGATTTGCACTAACTAGTAGGAG<br>GTCACCATCTCTTAAGTGATTCCTCCCAGACTATTTTAGCTCTCAGGGACAGCCAGGGCCCTCAACGATTCCCTAAGCATCTCTGGGAAAGTTTAC<br>ACATTACGCTGTTACAAGCAATTCATGTATGTGTGCATAGTGAAAACCTTTTCTCTTAAAACTCTTAATACTGAAGTTCCCCAAACCTTTGGAAAAT<br>GTACAAGTCACAGATGCTCCTGTGACTTGTTTTTCCCTTCGGGTGCGTCCTCAACCTTGCTTAAATAAACCTCTATTGAACGAGACCTGCATCAGT<br>CACTTTTCGGTTAACACTAGGTATTCAACGAATAGACTAGTCCCGCCGTTGTCTCCAGATATGATGGTGCCCTGACCCAAAGAAGCGAGATACTCC<br>TTCCCAGGGCCTCGCTCCCGAGCATGGGTTATTTGCACAAAACCCCGCAGGAAGCTTTTAGAGCCACAGTCGGCTAGGCTGGAGTCAGACTCTG<br>TTACCTCCTGACTCTGCCATCACGAGCAAGGAGCCTGCTTCCTCATTGGCAAAAGGGAATGACCAAGGCACCACCTTACAGGGTTGTGGCAACG<br>GGCTCATGGAATTATACATGCCAAGTGGTTATCACAGGGCCCGCCACTGGCGGAAGGTGAGACAATGTGGACGGCGAGGAAATGGGAGCATCGG<br>CTGGAAACCAGGGACGGAGATCGGGGATGGGGGGCGGGCAAGGGGACAGCGGAAAGGGTGGGCGAGGGATCCGTGTCCCAACCCGCCGAGTC<br>GCAGCTCTGCCCCTGCGAGCTTCCGCCGAGACCGCCCCCCGGGAAGCCGCGTTTCCTTCCTGCAGTCGCGGTCCGGCGTGGGGCACGGAGGGG<br>CTCGCGCTCCCAGAAGCCGGCAGGCCTTCCCGCGGGCGGGCCCGCAGCGCGCTCCGCCCCGGAAACACGCCCATGGCCGCCTTGGCGCGCTTT<br>CAAAC

Supplementary Table 8. Associations between E2F4 expression and clinicopathological features of 80 patients with gastric cancer.

Histopathological parameters	Total number (80)	Expression level of E2F4		*P*-value
		Low	High	
Age, years				0.797
<65	33	15	18	
≥65	47	20	27	
Gender				0.429
Male	51	24	27	
Female	29	11	18	
Tumor size				<0.001
<6	43	27	16	
≥6	37	8	29	
Lauren type				0.122
Intestinal type	73	30	43	
Diffuse type	7	5	2	
Depth of invasion				0.304
T1, T2	23	8	15	
T3, T4	57	27	30	
Lymph nodes metastasis				0.013
N0, N1	40	23	17	
N2, N3	40	12	28	
Distant metastases				0.005
Negative	58	31	27	
Positive	22	4	18	
TNM stage				0.029
I	11	9	2	
II	12	6	6	
III	33	13	20	
IV	24	7	17	
Degree of differentiation				0.849
Highly	43	17	26	
Moderately and poorly	37	18	19	
Histological grade				0.528
I	8	5	3	
II	38	16	22	
III	34	14	20	
Venous invasion				0.443
No	45	18	27	
Yes	35	17	18	
Nerve invasion				0.755
No	35	16	19	
Yes	45	19	26	

Supplementary Table 9. Prognostic factors on overall survival were analyzed by univariate and multivariate Cox's proportional hazards models in 80 patients with gastric cancer.

	Univariate analysis			Multivariate analysis		
	**HR**	**95% CI**	**P-value**	**HR**	**95% CI**	**P-value**
Age	0.225	0.458-1.392	0.799			
Gender	0.527	0.370-0.942	0.590			
Tumor size	2.414	1.033-5.641	0.042	2.224	1.161-4.262	0.016
Lauren type	1.557	0.949-2.555	0.08			
Lymph nodes metastasis	0.590	0.350-0.970	0.038	0.580	0.350-0.970	0.036
Distant metastases	3.440	1.372-8.625	0.008	3.345	1.682-6.648	0.001
Depth of invasion	1.131	0.461-2.771	0.788			
TNM stage	4.376	1.159-16.521	0.029	4.014	1.757-7.170	0.001
Degree of differentiation	0.847	0.416-1.724	0.647			
Histological grade	0.870	0.390-1.938	0.733			
Venous invasion	0.961	0.535-1.726	0.893			
Nerve invasion	0.970	0.471-1.996	0.934			
E2F4	1.075	1.789-4.800	0.001	0.730	1.205-3.573	0.008
DSCC1	1.114	1.893-4.902	0.001	0.098	0.483-2.518	0.012

HR, hazard ratio; CI, confidence interval.
